# Polymeric Nanocomposites and Nanocoatings for Food Packaging: A Review

**DOI:** 10.3390/ma11101834

**Published:** 2018-09-26

**Authors:** Cornelia Vasile

**Affiliations:** Physical Chemistry of Polymers Department, Petru Poni Institute of Macromolecular Chemistry (PPIMC), Romanian Academy, 41A Gr. Ghica Alley, RO 700487 Iasi, Romania; cvasile@icmpp.ro

**Keywords:** polymer, nanocomposites, nanocoatings, food packaging, risks, smart nanomaterials

## Abstract

Special properties of the polymeric nanomaterials (nanoscale size, large surface area to mass ratio and high reactivity individualize them in food packaging materials. They can be processed in precisely engineered materials with multifunctional and bioactive activity. This review offers a general view on polymeric nanocomposites and nanocoatings including classification, preparation methods, properties and short methodology of characterization, applications, selected types of them used in food packaging field and their antimicrobial, antioxidant, biological, biocatalyst and so forth, functions.

## 1. Introduction

Nanoscience and nanotechnology applications to the agriculture, food sector and food safety are relatively recent compared with their use in cosmetics and personal care products (60%), paints and coatings, catalysts and lubricants, security printing, drug delivery and pharmaceuticals, medical therapeutics and diagnostics, energy production, molecular computing and structural materials [1,2,3,4]. Over $17.8 billion worldwide investment was made in 2010 for research and development in nanotechnology and this will grow to $3 trillion by 2020. Food packaging sector counts for ~50% market value for all nanotechnology-enabled products. Annual growth rate is 11.65%. Nanotechnology is mainly applied in extremely high gas barriers packaging materials with antimicrobial properties and in nanoencapsulants for the delivery of nutrients, flavours, or aromas [5,6].

According to regulation (EC) = No 1935/2004 a good packaging should have the following functions: “to protecting the food from dirt or dust, oxygen, light, pathogenic microorganisms, moisture and a variety of other destructive or harmful substances. Packaging must also be safe under its intended conditions of use, inert, cheap to be produced, lightweight, easy to dispose or to reuse, able to withstand extreme conditions during processing or filling, impervious to a host of environmental storage and transport conditions and resistant to physical abuse” [7].

Nanomaterials are applied in packaging and food safety are in various forms such as: polymer nanocomposites with high barrier properties, intelligent packaging, nanocoatings, surface biocides, active packaging, silver nanoparticles as antimicrobial agents, nutrition and nutraceuticals, nanosensors and assays for the detection of food relevant analytes (gases, small organic molecules and food-borne pathogens) and bioplastics. 

Main recent development areas in packaging field are:

(I) *Improved packaging* which offers improved mechanical properties as flexibility, enhanced barrier properties against water, gases, taint, durability, temperature/moisture stability, and so forth;
(II) *Active/bioactive food packaging* offers antimicrobial, antioxidant or biocatalytic functions. It can be obtained by the incorporation of active/bioactive compounds into matrices used in existing packaging materials, or by the application of coatings with the mentioned functionality through physical or chemical surface modification. Active packaging is applied in food packaging, pharmaceuticals and consumer goods in order to improve shelf life, safety, or quality of packaged foods. Coating option is advantageous because the bulk properties of the packaging materials are preserved almost intact by using a minimum amount of active agent required to impart efficacy and therefore also cost is reduced.
(III) *Smart/intelligent packaging* as a promising area for active packaging coating is developed by manufacture of nano(bio)sensors which can indicate quality of foodstuffs, of nano(bio)switch to release preservatives and nano-coatings as antimicrobial, antifungal, antioxidant, barrier coatings, external stimuli responsive materials and self-cleaning food contact surfaces. Intelligent inks such as nanoparticles and reactive nanolayers allow analyte recognition at nanoscale. Printed labels are applied to indicate: temperature, time, pathogen, freshness, humidity, integrity [8]. Smart packaging may monitor various parameters such as: temperature, oxygen, pH, moisture and so forth [9,10,11] of packaged products.

## 2. Polymeric Nanocomposites

Polymeric nanocomposites are mainly obtained by dispersing of nanoscale filler into a polymeric matrix. 

Polymers commonly used in food packaging as either matrices or substrates for bioactive coatings are synthetic undegradable as low density and high density polyethylene (LDPE and HDPE), polypropylene (PP), polyethylene terephthalate (PET), ethylene vinyl alcohol (EVOH) copolymer, polyamide (PA), polystyrene (PS) and degradable ones as polyhydroxyalkanoates (polyhydroxybutyrate (PHB), poly(hydroxybutyrate-*co*-hydroxyvalerate) (PHBV)), poly(lactic acid) (PLA), polycaprolactone (PCL), polyvinyl alcohol (PVOH) and biopolymers as polysaccharides and proteins with critical issue on water resistance, migration and permeability and so forth These properties may be improved by adding reinforcing compounds (nanofillers), forming bionanocomposites [12]. Because of the different characteristics, their application in food packaging is specific. For example, HDPE is used as milk bottles and bags, LDPE for trays and general-purpose containers, PP has a high melting point, being ideal for hot-fill liquids, films and microwavable containers, while PET is clear, tough and has good gas and moisture barrier properties, so it is used for soft drink bottles, beverages and mineral waters, carbonated drinks and so forth [13]. While synthetic polymers generate huge waste quantity and have an environmental negative impact, by using degradable polymers these problems are avoid, because they are biocompatible and safe to use. However, most degradable polymers are in the research stage or their cost is high, some food packaging based on PLA (the cheapest bio-based material) and poly(butylene adipate-*co*-terephthalate) (PBAT) based packaging is used in Europe, Japan and North America [14,15] 

Moreover, recently, a series of biodegradable nanocomposite films based on PBAT and reinforced with an organophilic layered double hydroxide OLDH (0.5−4 wt %) nanosheets were scale-up fabricated. Xie et al. reported such materials with “outstanding thermal, optical, mechanical and water vapour barrier properties a 37% reduction in haze and a 41.9% increase of tensile strain at break better than the pure PBAT film and commercial polyethylene packing materials” [16].

The active agents used in active packaging and coatings include antimicrobials, antioxidants and enzymes which control microbial growth, inhibit oxidative degradation reactions and offer a targeted biocatalysis that maintains food safety and quality and controls spoilage.

**Figure 1:**
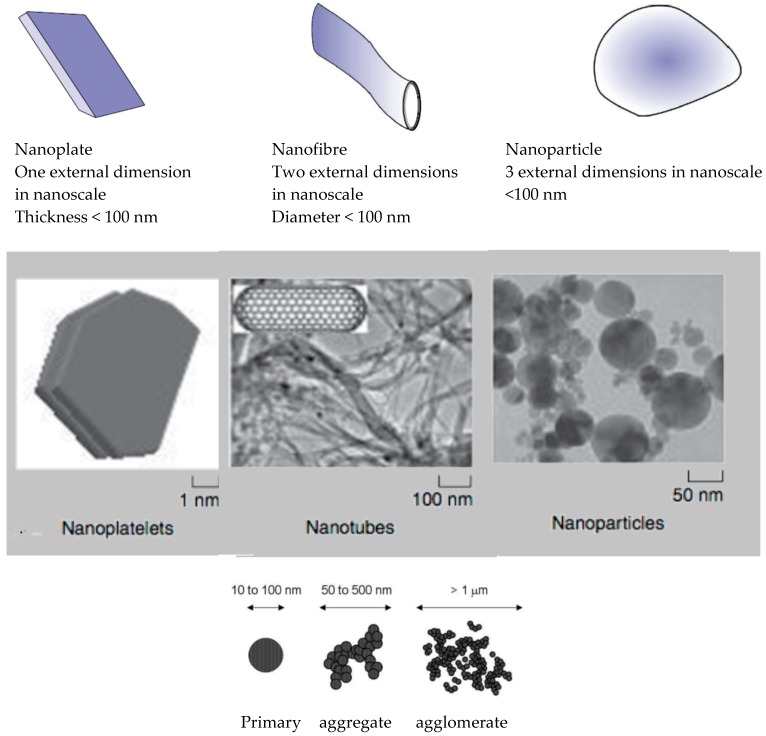
Representative images for nanofillers for nanocomposites. adapted from [17,18,19].

The most used nanofillers and nanoreinforcements are: clay montmorillonite (MMT) and kaolinite and silicate nanoplatelets (two-dimensional layers, which are 1 nm thick and several microns long), silica nanoparticles, carbon nanotubes, graphene nanosheets, silver, zinc oxide, titanium dioxide, copper and copper oxides, starch nanocrystals, cellulose nanofibres and nanowhiskers, chitosan and chitin whiskers and others. In respect with their geometry isodimensional nanoparticles have three nanometric external dimensions while nanotubes or whiskers are elongated structures with two external dimensions on nanometre scale and the third is larger (Figure 1) [17,18,19]. Other classification is zero-dimensional, one-dimensional, two-dimensional and three-dimensional nanostructured materials [20]. Nanofibres may be also known as nanowire (electrically conducting nanofibre), nanotube (hallow nanofibre) and nanorods (solid nanofibre) [12].

When only one dimension is in the nanometre range, the composites are known as polymer-layered silicate nanocomposites [21,22]. The role of nanofillers and nanoreinforcements in composites consists in the enhancement of the mechanical, thermal (glass transition, melting and degradation temperature) and barrier properties by increasing path length for gas diffusion, changes in surface wettability and hydrophobicity and so forth For food packaging they may offer antimicrobial activity, oxygen scavengers, enzyme immobilization, biosensing and so forth Nanocomposites usually represent significant changes in properties at low loads <5 wt % of nanofillers and <2% volume. Also, nanocomposites improve the stability of sensory properties, such as flavour, better maintenance of colour and texture, increased product stability through the food chain and less spoilage. It is well-known that the interfacial region in nanocomposites is extremely large, therefore the interaction of the polymer with the nanoparticles is strong and this will change the polymer mobility and relaxation dynamics, decreases chain mobility due to nanomodification and that means an increase in Tg [23]. 

Nanocomposites may improve both thermal and environmental dimensional stability. Two factors are considered responsible for improving the thermal stability in thermoplastic nanocomposites with respect to the neat matrix: (i) the chemical composition and morphology of the nanocomposites is different from that of the pristine polymer; (ii) the thermal motion of the polymer molecules is restricted by the filler nanoparticles. Incorporation of nanofillers in thermoplastics usually led to a marked increase in the melt viscosity, especially in the range of low frequencies. 

Several types of polymer nanocomposites are known depending on the nanofiller used as mentioned above. Examples include UV absorbers to prevent UV degradation of plastic polymers (e.g., nano-titanium dioxide, iron oxides, silica, alumina), titanium nitride (TiN) used to improve the strength of materials, nano-calcium carbonate-polymer composites, biodegradable starch and/or polylactic acid with various nanoclay composites, gas barrier coatings and so forth [24,25,26,27,28].

### 2.1. Preparation Methods

The preparation methods for polymer nanocomposites should assure a good dispersion of nanofiller into matrix to obtain intercalated or exfoliated structures [29,30,31,32,33]. The exfoliated nanocomposites are also classified as ordered and disordered exfoliations. Partial exfoliation is an intermediate morphology between intercalation and exfoliation. Significant variations in physical and mechanical properties of polymer nanocomposites are directly correlated with the differences in morphology [34].

There are two main strategies to obtain nanostructures and consequently polymer nanocomposites and nanocoatings—Table 1—so called “top down” or ex situ synthesis (attachment of nanoparticles to the polymer matrices prepared into different step) and “bottom up” or in situ synthesis methods (e.g., in situ polymerization, spin coating, casting) [35]. In the “top-down” approach the nanoparticles are produced in a separate step and then they are dispersed in polymer (direct mixing of the filler into a polymeric matrix, ball milling and application of severe plastic deformation.), melt, or monomer solution which is then polymerized or nanostructures are generated by mechanical disintegration (milling) of the previous prepared material. In the “bottom-up” approach, the nanostructures are built up in a chemical process consisting in generation of nanoparticles inside polymer matrix or monomer polymerizing solution by chemical, thermal or photolytic decomposition of some precursors. Bottom-up approaches may be in gas-phase or in liquid phase such as: sol-gel processing, chemical syntheses, spraying, plasma or flame sputtering, spinning to make thin polymer fibres, chemical vapour deposition (CVD), laser pyrolysis, atomic or molecular condensation, electrodeposition, supercritical fluid synthesis, and so forth [36], while among the top-down techniques it is possible to include the energetic mechanical milling, sonication and so forth.

The preparation method determines both the concentration and distribution of the nanofillers and nanoreinforcements into the polymer [37]. 

The in-situ polymerization technique was widely used to obtain different kinds of nanocomposites such as those based on poly (vinyl acetate), nylon 6, in situ synthesis of poly(urethanes) PU in in dimethylformamide (DMF), emulsion polymerization of styrene in water and many others [38,39].

*Solution mixing* or *solvent casting* involves the vigorous stirring or ultrasonication of the nanoparticles in a polymer solution before casting in a mould and then evaporating the solvent. Both water and organic solvents can be used to prepare nanocomposites with either thermoplastics or thermosets. The removal of organic solvent after casting has environmental implications. The polarity of the solvent is a critical characteristic which influences the intercalation of the polymer into the space between the clay platelets. Nanocomposites are prepared by *emulsion polymerization* of acrylates in silica sols. By using this technique, it is possible to achieve a homogeneous distribution of silica nanoparticles into polymer at silica content up to 50% in the nanocomposites.

At industrial scale of the *extrusion* or *melt processing*, the compounding of the components of a nanocomposite material is performed in a single or twin-screw extruder where the mixture of polymer and the nanofiller is a melt state. The shear and elongational stress applied with mixer during process help to break the filler agglomerates and uniformly dispersing them into the polymer matrix. As end products, films and other items can be formed from blend by profile injection moulding, extrusion, blow moulding. The intrinsic lack of thermal stability of many active/bioactive compounds which can be lost through degradation and evaporation during the heat transfer and high shear that also could degrade both the polymer and the nanofillers (as carbon nanotubes) limit the applicability of this preparation method. The homogeneous distribution of active agents into matrix is difficult. Processing at high shear or sonication techniques are used to deaggregate or exfoliate the clusters and to increase the surface area exposed to the polymer. Polymers able to interact with nanofillers give a good dispersion. 

*Shear mixing*: Low-shear mixing or high-shear mixing can be used for incorporating solid nanoparticles into a liquid polymer. Under these conditions and if the nanoparticles are compatible with the selected polymer, the mixing will disrupt the nanoparticle aggregates and disperse the polymer matrix into the nanoparticle layers or onto the nanoparticle surfaces. The dispersion degree of the nanofiller and the filler/matrix interaction can be generally improved by (i) using surface-treated filler to reduce aggregation phenomena and (ii) incorporating a compatibilizer or a tensioactive agent within the polymer matrix (such as maleic anhydride grafted polypropylene). 

LBL and electrospinning techniques are described in the nanocoatings section. 

### 2.2. Types of Polymer Nanocomposites Used in Food Packaging

Some types of nanocomposites used in food packaging are given in Table 2 [12,33].

#### 2.2.1. Montmorilonites (MMT) Containing Nanocomposites

The polymer–clay morphologies are classified in (1) tactoid, (2) intercalated and (3) exfoliated. In the *tactoid structures* due to the poor affinity of nanoclay with the polymer, the interlayer space of the clay gallery will not expand, the components do not mix each other and nanocomposites are not formed. In *intercalated structures* a moderate affinity between polymer and clay exists hence they are characterized by moderate expansion of the clay interlayer but the shape remains unchanged, polymer chains are able to penetrate the basal spacing of clay, mixing of components being homogeneous. A high affinity between polymer and nanoclay promotes separation of the clay clusters into single sheets within the continuous polymer matrix resulting a homogeneous dispersion as the *exfoliated structures* which determine important changes in properties of the nanocomposites in respect with those of neat polymer. 

Generally, MMT behaves as an effective reinforcement/filler, offering a high surface area and large aspect ratio. However, in many cases the compatibility with the matrix should be improved both by organophilization of the filler, the use of surfactants or compatibilizers. By these ways better exfoliated structure and better mechanical properties are achieved for nanocomposites. Clay layers constitute a barrier to gases and water, forcing them to follow a tortuous path, nanocomposites showing improved oxygen and water vapour barrier properties and some of them as Cloisire 93A, Cloisite 30B and Dellite High Pressure Sodium HPS offer also antimicrobial activity against both Gram-positive and Gram-negative bacteria, excepting fungus *Candida albicans* [40]. In many cases the mechanical properties are improved, glass transition and thermal degradation temperatures increase but some nanoclays decrease transparency of the films [12].

MMT containing nanocomposites show many advantages in respect of neat corresponding polymeric matrices; they are lighter, stronger, more heat-resistant, offer improved barrier against gases, moisture and volatiles materials, antimicrobial properties and so forth. They are produced by Nanocor Inc. (Arlington Heights, IL, USA) and Southern Clay Products, Inc. (Gonzales, TX, USA), Mitsubishi Gas Chemical (New York, NY, USA) and other units using as matrices Nylon-6, PE, PET and EVOH. Films, PET bottles and multilayer films from nanocomposites are obtained to prolong the shelf life of a variety processed meats, cheese, confectionery, cereals and boil-in-bag foods and so forth [8,68,69].

#### 2.2.2. Bionanofibrils

*Biopolymer nanofibrils* with dimensions of angstrom to hundreds of nanometre scales arise from renewable resources, are widely available at low-cost, offering exceptional biocompatibility, biodegradability, flexibility and the availability of multiple reactive sites for introducing novel functionalities. Because of the natural origin and well-defined supramolecular assemblies and geometries they exhibit exceptional mechanical properties mainly a unique combination of strength and toughness. Their biological functions allow the interactions with the surrounding environment performing a variety of specific functions in living systems and also serving as building blocks stabilized by non-covalent interactions in material science. The nanofibrillar biopolymers are potential candidates for high-performance and functional bionanocomposites as strong, sustainable, stimuli responsive and biocompatible materials for a wide range of applications such as environmental, energy, food packaging, optical and biomedical [70,71]. The combination of biological and synthetic components frequently shows synergistic effects because of strong interfacial interactions that significantly enhance the structural performance and facilitate added functionalities of nanocomposites. In some bionanocomposites, novel synthetic nanoparticles (graphene, carbon nanotubes, mineral nanoparticles, metallic nanoparticles and so forth) were efficiently combined with biological components to achieve superior electrical and thermal conductivity, controlled gas barrier properties, complex actuation and unique optical properties [72]. The biopolymer nanofibrils as cellulose nanofibrils (CNF), chitin nanofibrils (ChNF), silk nanofibrils (SNF) and collagen nanofibrils (CoNF) are most abundant nanofibrils in nature. The nanocellulose and silk nanofibrils, are representatives of classes of polysaccharides and polypeptides [73]. 

Both top-down and bottom-up strategies have been developed to exfoliate and regenerate bionanofibrils. 

Nanocellulose is a term referring to nano-structured cellulose. This may be either cellulose nanocrystal (CNC), cellulose nanofibers (CNF) also called microfibrillated cellulose, or bacterial nanocellulose, which refers to nano-structured cellulose produced by bacteria (BC). They are renewable abundant raw materials derived from wood and plants. As biodegradable fibre-reinforcement for polymeric bionanocomposites, they also offer excellent tensile properties, non-toxicity, high thermal conductivity and optical transparency as indispensable requirements for advanced food packaging. 

*Cellulose-based nanoreinforcements* (as cellulose microfibrils or nanofibers and microcrystalline cellulose). Cellulose microcrystals may contain also amorphous areas. Nanofibres have nanosized diameters (dimension range of 2–20 nm, depending on the bionanofibril origin) and lengths in the micrometre range. Whiskers are crystalline parts and are known as nanocrystals, nanorods, or rod like cellulose microcrystals with lengths ranging from 500 nm up to 1–2 μm and about 8–20 nm or less in diameter and high aspect ratios. Microcrystalline cellulose has 200–400 nm in length and an aspect ratio of about 10 [12]. Their dimensions affect the properties of nanocomposites. Nanocellulose can be extracted via enzymatic pre-treatments, tempo oxidation and chemical extraction, while CNC are extracted generally via acid hydrolysis with sulfuric acid [74,75,76]. 

Polysaccharide nanoparticles as aqueous suspensions hydrosoluble (or at least hydrodispersible) or latex-form polymers are obtained. The dispersion of nanocrystals into non-aqueous media is also possible using surfactants or chemical grafting. These are other possibilities for processing of the nanocomposites. The surface of the polysaccharide nanocrystals is covered with reactive hydroxyl groups, which provide the possibility of their extensive chemical modification using grafting agents bearing a reactive end group and a long compatibilizing tail [30]. The surface chemical modification of the cellulose biofibres leads to improved interfacial adhesion between the fibres and the matrix, resulting in enhanced mechanical properties and thermal stability. CNC coatings are also transparent, nontoxic and sustainable. CNCs are highly crystalline and easily dispersed in water, therefore the structure can be controlled to eliminate free volume and to improve barrier properties of the materials. They offer to coatings a higher density and packing that reduces diffusion pathways and drastically improves oxygen, carbon dioxide and water vapour permeability properties which are similar with those of EVOH commercial food packaging material. Their use is also associated with biodegradability and sustainability [77]. *Bacterial cellulose* (*BC*) *nanofibrils* randomly-oriented as web-like form show full potential as reinforcement. To orient the nanofibrils a controlled stretching of BC hydrogel was performed by Rahman and Netravali [78,79]. They obtained a BC-reinforced soy protein soy protein isolate (SPI) by vacuum-assisted SPI resin impregnation into BC hydrogel and then stretching the resin impregnated BC hydrogel. Aligned BC nanofibrils were produced by cultivating them inside polydimethylsiloxane (PDMS) tubes followed by wet-stretching. Due to higher BC nanofibrillar orientation, the stretched BC-SPI green composites showed significant improvement in their tensile properties. Cellulose nanofibrils were produced from commercial bleached cellulose pulps after 30 passages in a SuperMasscolloider Grinder (MASUKO SANGYO CO., Kawaguchi, Saitama, Japan), or using mechanical processes [80]. Films were prepared by a casting method. BC-reinforced PLA showed enhanced barrier and mechanical properties [81].

*Hybrid nanocomposites*, where two different nanoparticles CNC/metallic nanoparticles CNF/clay and CNC/clay) are incorporated into the polymer have been also obtained. [82] PLA-based nanocomposites with CNF, 1–5% nanocrystalline cellulose (CNC) and 1–5% nanoclay (Cloisite™ 30B) for food packaging use led to up to a 90% reduction in the oxygen transmission rate (OTR) and 76% reduction of water vapour transmission rate (WVTR), significant increase in thermomechanical resistance and increased crystallisation kinetics. [83,84] The incorporation of nanoparticles will increase the tortuosity within the material and that this increases barrier properties.

The corona treatment leads to the quality improvement of fabrics and composites. It was applied with good for surface modification and physical and mechanical properties of nanofibril containing films useful for printing and packaging materials such as eucalyptus and pinus nanofibril films [85]. After the corona discharge and subsequent exposure to oxygen from air hydroxyl, carbonyl and other functional groups are formed by breaking C–C bonds and interaction with active species from plasma (see below). After such treatment, the Eucalyptus nanofibril films showed increased tensile strength due to their higher crystalline index and nanofibril dimensions. 

*Chitin nanofibrils* were directly extracted from crab shells through a green deprotonation-assisted liquid exfoliation procedure. By aqueous re-dispersibility after freezing/air/thermal drying, hybridization with other two-dimensional nanomaterials, (e.g., graphene and transitional metal dichalcogenides) was performed. Plasticized PLA with 1 and 5 wt % chitin nanocrystals (ChNC) nanocomposite after orientation, showed a ‘shish-kebab’ morphology in the drawn tapes. Singh et al. reported improved mechanical and thermal properties. The tensile strength increased and the elongation at break increased from 5% to 60% for the nanocomposite with 5 wt % ChNC and a draw ratio of 3 because of the synergistic effects of the ChNC in the nanocomposite and their alignment of the ChNC together with the polymer chains induced by the solid-state drawing [86]. 

*Silk fibres.* A variety of artificial spinning methods have been applied to produce regenerated SNFs. Ling et al. developed a bioinspired spinning method [87]. They prepared nematic silk microfibril solution, highly viscous and stable by partially dissolving silk fibres into micro-fibrils. The hierarchical structures in natural silks was maintained in solution which was spun into regenerated silk fibres by direct extrusion in the air. In this way, polymorphic and hierarchical regenerated silk fibres with physical properties beyond natural fibre construction were generated. By further functionalization with a conductive silk/carbon nanotube coating, responsive materials to changes in humidity and temperature can be produced. 

The following surface modification were applied: Poly (ethylene glycol) PEG-grafted rod-like cellulose microcrystals [88], alkenyl succinic anhydride acylating the surface of cellulose nanocrystals, crab shell chitin whiskers [89], waxy maize starch nanocrystals [90] and tunicin whiskers used then to reinforce some plastics [91,92] their performance depending on type of matrix. 

*Starch nanocrystals* show platelet morphology with thicknesses of 6–8 nm. Their incorporation lead to improve some properties of PVOH [93] and pullulan [94]. 

#### 2.2.3. Other Types of Nanofillers

*Carbon nanotubes* (*CNT*)*.* Tensile strength/modulus of PVOH [95], PP [96], PA [97] are improved by addition of CNTs.

*Silica nanoparticles* (*nSiO_2_*) improve mechanical and/or barrier properties of PP [98], mainly when maleic anhydride grafted polypropylene (PP-g-MA) is used as a compatibilizer [99], of PVOH [100] or of starch matrix [101]. 

*Haloysite* (HAL) represents a class of natural, non-toxic, non-swelling silicates, stable even at very high temperatures and show a good encapsulation and release capacity for bioactive compounds (anticorrosion, antimicrobial, drugs, flame retardant, microcrack self-healing). They have a wide variety of applications including also as fillers and reinforcements for polymers. The HAL particles can adopt a variety of morphologies, as elongated tubules known as haloysite nanotubes (HNT), short tubular, spheroidal and platy particle shapes, the first one being the most common [102]. HAL can be incorporated into different polymers to obtain non-degradable and degradable nanocomposites with versatile properties some of them being attractive for potential uses in food packaging [103]. The HNT-incorporated polymer nanocomposites can be prepared by various processing routes such as melt blending, melt spinning, water-assisted extrusion process, methods involving hydrogen bonding and charge transfer mechanism, solution casting, co-coagulation or co-curing processes, in situ or emulsion polymerization techniques for nondegradable nanocomposites and solution casting, coagulation, electrospinning for HNT-containing degradable nanobiocomposites. HNTs containing nanocomposites exhibit remarkable mechanical performance and their properties can be tailored by HAL surface modification [104], by nanocomposites composition and by the preparation methods optimization. HAL nanofillers act as ethylene gas absorbers. This gas causes softening and aging of fruits and vegetables; limits the migration of spoilage-inducing gas molecules within the polymer matrix. Active food packaging materials as HNT/PE nanocomposite films showed higher ethylene scavenging capacity and OTR and WVTR than neat PE films. Due to their ethylene scavenging activity and good water vapour and oxygen barrier properties, the nanocomposite films slow down the ripening process of bananas and retain the firmness of tomatoes and also slowed down the weight loss of strawberries and aerobic bacterial growth on chicken surfaces. HNT/PE nanocomposite films contribute to food safety improving the quality and shelf life of fresh food products [105]. Starch/halloysite/nisin nanocomposite films inhibited activity of *Listeria monocytogenes*, *Clostridium perfringens* and *Staphylococcus aureus* in skimmed milk agar being active and useful barrier to control food contamination [106]. HNTs loaded with lysozyme, as nano-hybrid antimicrobial was incorporated into a poly (ε-caprolactone) (PCL) matrix at 10 wt % and then the films were submitted to a cold drawn process. The films exhibit a controlled release of lysozyme being for specific active packaging requirements [107]. 

*Graphene* as atomically thin carbon sheets improve physical properties of host polymers at extremely small loading. Nanocomposites containing graphene are obtained by top-down strategies starting from graphite oxide, solvent- and melt-based strategies [108]. Such nanocomposites offer extremely high barrier performance at even very low graphene loadings, which is interesting for food packaging [109].

*Metallic nanoparticle* incorporated into food contact polymeric matrices enhance mechanical and barrier properties, prevent the photodegradation of plastics and are effective antimicrobials (as salts, oxides and colloids, complexes) such as AgNP [110]. Copper, zinc and titanium NP are also useful in food safety and technology. Copper is an efficient sensor for humidity and titanium oxide has resistance to abrasion and UV-blocking performance [111].

*Lignin nanoparticles* were tested for their antioxidant, antimicrobial activities and potential biological being effectively used as active agents towards different pathogen strains when is combined with PVA, chitosan CH, natural polymers and so forth [112,113]. 

*Multifunctional nanocomposites* contain usually a mixture of micro- and nanostructured materials such as essential oils, natural extracts and metal nanoparticles with antimicrobial/antioxidant agents/biological functions and they are also interesting for food contact materials.

#### 2.2.4. Bioplastics–Biopackaging 

Increased use of the synthetic packaging materials (as films, bottles, trays and so forth) has led to serious ecological problems due to their non-biodegradability and waste accumulation. Biopolymers derived from natural resources as plants (starch, cellulose and their derivatives, proteins), animal (proteins, polysaccharides) and microbial products, such as polyhydroxybutyrate have been extensively studied in the bio-based nanocomposites field [114] because of their good biocompatibility, degradability [115] and recyclability [116]. The starch-clay biodegradable nanocomposites were investigated for several applications including food packaging. Some restrictions are encountered on the use of biodegradable or natural polymers in food packaging since they have poor barriers and mechanical properties, high permeability to gases, such as oxygen and water vapour because of their hydrophilic nature with a few exceptions, such as caseinates, which show excellent barrier performance, even better than PET [117]. Nanoreinforcements can improve barrier properties and have a positive impact on the oxidation stability, thermal and mechanical characteristics and eventually bionanocomposites show the good biodegradability comparing with conventional polymeric matrices. Polymer cross-linking and graft copolymerization of natural polymers with synthetic monomers/polymers are other alternatives of promising values in biodegradable packaging. The complete replacement of synthetic polymers with degradable ones is almost impossible to achieve and even unnecessary, at least for specific applications the use of biodegradable polymers is desirable. No doubt, In the future biopackaging will be increasingly developed [46,118]. 

*Protein-based nanocomposites.* In commercial applications, the following animal derived proteins used are: casein, whey protein, collagen, egg white and fish myofibrillar protein. The plant-based proteins also under large scale application consideration include soybean protein, zein (corn protein) and wheat gluten. Compared with non-ionic polysaccharide films, protein films, due to their more polar nature and more linear (non-ring) structure and lower free volume offer better oxygen barrier properties and lower water vapour permeability [119]. The transparent films based on whey protein show good oxygen barrier and TiO_2_ or ZnO ware added to form nanocomposites with improved antimicrobial properties [120,121,122] used to fabricate food-grade, biodegradable packaging materials. Chen et al. and Yu et al. showed that the addition of small amounts (<1 wt %) of TiO_2_ nanoparticles significantly increased the tensile properties of whey protein film (from 1.69 to 2.38 MPa). Soy protein nanocomposite films exhibited reduced water vapour permeability, improved elastic modulus and tensile strength compared with neat polymer [123,124]. Zein as biodegradable polymer is used in the food industry as a coating agent [125]. It is less water sensitive than other biopolymers but shows high water vapour permeability and low tensile strength when compared with commodity polymers. To ameliorate its inherent brittleness, plasticizers may be used.

#### 2.2.5. Degradability and Recyclability of Nanocomposites

Bionanocomposites (BNC) can be recycled/valorised or treated together with other organic wastes in composting facilities and produce compost, as valuable soil conditioners and fertilizers and so forth [126]. They are high-performance biodegradable materials, based on plant, animal and other natural materials, therefore these are safely decomposed into CO_2_ consumed during plant photosynthesis, water and humus through the activity of microorganisms [127]. This behaviour refers to with BNC with degradable polymeric matrices like poly(lactic acid) (PLA), poly(butylene adipate-*co*-terephthalate) and thermoplastic starch which undergo degradation after various time period. Different biodegradation conditions can be considered: hydrolytic, composting, enzymatic, according to the final applications and the post-use of the new developed materials [128].

Combining the individual advantages of starch/synthetic or natural polymers and nanoparticles (cellulose nanocrystals, clay, TiO_2_, layered silicate and so forth) starch-based completely biodegradable materials with potential applications in food industry as edible films were prepared. [129].

The effect of the nanofillers on degradability and compostability of bioplastics depends on their type. PLA/Laponite biocomposites showed the greatest microbial attachment on the surface (biofilm is formed), while PLA/ organo-montmorillonite (OMMT) had the lowest biofilm formation because of the inhibitory effect of this NP during biodegradation test. Nanoclays influence the polymer bacterial degradation due to the affinity of the bacterium towards clay. The addition of nanoclays during composting process increases the PLA degradation rate due to the presence of hydroxyl groups from the silicate layers of these clays. As an example, due to the hydrophilic nature of nanocellulose, the cellulose nanocrystals (CNCs) increased the disintegrability rate of PLA.

PLA bio-nanocomposite films containing nanoclay with organo-modified (with methyl, tallow, bis-2-hydroxyethyl, quaternary ammonium montmorillonite), Halloysite nanotubes and Laponite^®^ RD, MMT showed a significantly higher mineralization of the films in comparison to the pristine PLA, mainly attributed to the reduction in the PLA lag time. PLGA/CNT films were degraded by hydrolytic degradation [130]. CNTs insignificantly modifies the kinetics and the mechanism of the hydrolytic erosion with respect to the neat PLGA, while CNTs functionalized with carboxylic groups accelerated the hydrolytic degradation and the weight loss of the PLGA matrix of the nanocomposites.

MWCNTs reduce the biodegradation rate of PCL in the presence of *P. aeruginosa*, systematically as the CNT loading increased from 0.1 to 10% *w*/*w* [131].

## 3. Polymer Nano-Coatings in Food Packaging

A coating is defined as a coherent layer formed from a single or multiple applications of a coating material to a substrate. Nanocoatings are ultra thin layers on the nanoscale <1–100 nm thick built-up onto surfaces [132,133]. Nanocoatings do not modify the surface topography, they do not fill in defects or make a smooth surface like a paint does, and they also do not stand up to abrasion and wear. They are used to impart a particular chemical or physical function(s) to a surface as gas-barrier coatings, hydrophilic/hydrophobic or oleophobic properties, improve corrosion resistance and enhance insulating or conductive properties [134].

**Figure 2:**
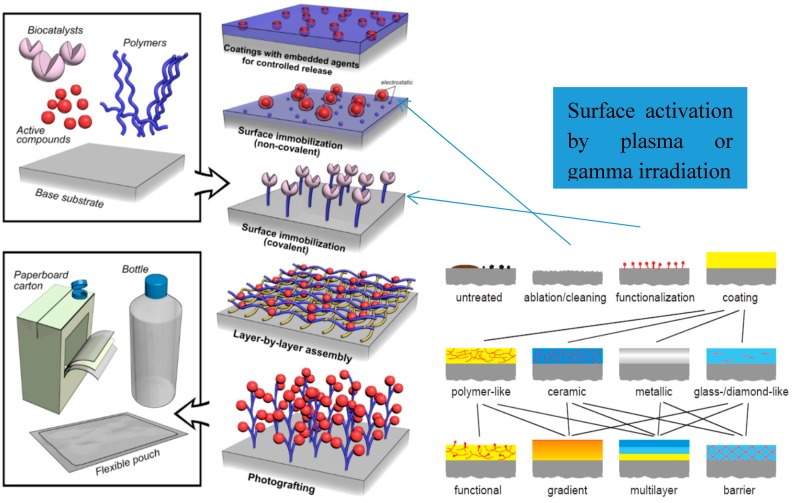
Summary of coating technologies (adapted from [135,136,137,138,139,140]).

Nanocoatings can be built up with thickness of one molecular or multiple molecular layers. Nanocoatings are applied to various substrates such as metals, glass, ceramics, polymers and so forth (Figure 2) [135,136]. Some nanocoatings are polymers, either polymerized in-situ or prior to application. “*Smart coatings*” are coatings with multifunctional additional functions some also assuring thermal insulation, controlled release of active ingredients or self-healing functions. 

Additives are added to a coating material in very small quantities. They can modify a large variety of properties, for instance, flow behaviour, surface tension, gloss, structure, UV and weather resistance. Depending on the desired function, nanotechnology-based functional coatings uses some nanomaterials as in the case of antimicrobial coatings containing silver, titanium dioxide, zinc oxide, some organic bioactive agents as polysaccharides, proteins, spice and herb extracts as essential and vegetable oils, bacteriocins (ex: Nisaplin^®^, nisin, pediocins), organic acids for food packaging or hygienic surfaces. Nanomaterial-containing coatings offer high performance materials and better processing properties than conventional coatings (e.g., increased indentation resistance, high elasticity, fast drying, no expansion after contact with water, high water vapour permeability). 

### 3.1. Types of Nanocoatings

Several types of nanocoatings are known in food packaging applications as: nanocoating inside package, outside package, sandwiched as a layer in laminated multilayer packaging films, polymer with nanocoating with high barrier properties, edible coatings and films on a wide variety of foods which serve as moisture, lipid and as gas barriers [137], vacuum deposited aluminium coatings on plastic films, coating of the surfaces of glass food and beverage containers (bottles, jars) with organosilanes, nanosurface biocides and so forth 

Nanosilica-coated films onto PET, Nylon and so forth, replace polyvinylidene fluoride (PVDF) coated films and oxide evaporated films, for processed meat products (beef meta, sausage, ham and so forth, fresh food like rare fish, sushi, dried fish and so forth, bakery, sandwiches, snack, candy, nut products with high fat) and so forth [28], offering oxygen and moisture barrier and aroma preservation increased self-life and decreased production cost, good printability and laminating machinability, are eco-friendly, no dioxines are formed during incineration, excellent mechanical and optical properties (retaining bulk properties of base film).

*Nano surface biocides* (using as nanoparticles: nanosilver as metallic Ag, AgNO_3_, ZnO, TiO_2_, MgO) are obtained by incorporation such nanomaterials with antimicrobial properties onto packaging surface. Such food contact nano surface containing biocides maintain the hygienic conditions when applied to reusable food containers such as boxes and crates and inside liners of refrigerators and freezers. 

The most known coating technologies are immobilization using covalent (non-migratory) and non-covalent (migratory), layer-by-layer deposition, photografting chemistries and embedding for controlled release, schematically represented in Figure 2 [135,136,137,138,139,140]. 

In coating technology which uses the embedded agents for controlled release, the active agent is intended to migrate to the packaged good (embedding, non-covalent immobilization, some layer-by-layer deposition techniques) while in non-migratory technologies, the active agent is intended to remain stable in the packaging matrix (in covalent immobilization, some layer-by-layer deposition techniques, photografting). Some techniques to prepare nanocoating are similar with those for nanocomposites obtaining, some are adapted and other are specific for this purpose. In some cases, the nanocoatings application required specialized equipment and complex procedures, such as, chemical vapour deposition or plasma spray techniques. 

The active agents are expected to migrate and exert their specific antimicrobial, antioxidant, biocatalytic, or nutraceutical functions within a packaged food. Active compounds are incorporated into polymeric materials by extrusion/blending and pip-coating techniques or solution casting.

### 3.2. Coatings Procedures

#### 3.2.1. Solution Casting

Solution casting consists of dissolving the polymer in a suitable solvent and simultaneously incorporating the active compound of interest, followed by pouring this mix solution onto an inert surface. By the solvent evaporation, the films with desired functionality (antimicrobial, antioxidant, pharmaceutical, biocatalytic and so forth) result. It is mainly used at laboratory scale and can be translated at industrial scale by coupling with a wet-coating station and roll-to-roll system [141]. However, it has some limitations both for practical and commercial application because the most of the interesting polymers used in food packaging dissolve only at high temperatures and in certain organic solvents [142], which will affect both the stability and effectiveness of many active/bioactive compounds of interest. Therefore, the incorporation of active compounds through solution casting has mainly applied when biodegradable polymers dissolved at milder temperatures, such as poly(lactic acid), poly(butylene adipate-*co*-terephthalate) and cellulose derivatives, PVOH and so forth [143]. Butnaru et al. obtained films by casting and dried in a vacuum oven with controlled temperature using solutions of highly viscous chitosan from crab shells where food grade vegetable oils (clove, thyme and rosehip seeds oils) were incorporated were homogenized by ultrasonication [144]. 

#### 3.2.2. Extrusion

In commercial extrusion, the active compounds with polymeric materials in melted state, in a single or twin screw extruder by heat transfer where are incorporated a blend is obtained from which films can be formed. It also has serious limitations due to the intrinsic lack of thermal stability of many active compounds as it was already mentioned above, which can be lost either through degradation and evaporation. During the heat transfer involved in these unit operations the homogeneous distribution of active agent into matrix is difficult. The active compound release is controlled by packaging material characteristics such as the degree of affinity between it and the matrix, morphology and porosity and also by medium temperature. Multilaminated system in which the layer that harbours the active compound is covered by an adjacent layer could act as a barrier slowing release rate. In such case the bulk properties as tensile and thermal are changed therefore the coating technique is preferred. 

#### 3.2.3. Sol-Gel Procedure

Sol-gel procedure is extensively used in obtaining of nano-based coatings. A viscous colloidal-sol is applied to a surface by dip, spray or spin coating processes onto surface. The thickness of the obtained layers varies from a few nanometres thick to 0.5 and 3 µm. The process occurs at low temperature, in a short time, is low energy consuming and more important without disruption of the structure or functionality of biocomplexes or organic aggregates, the nanocoating as a gel may exhibit a porous 3D network [145].

#### 3.2.4. Spraying Solution with Compressor Gun

There several types of sparaying guns as manual and automatic spray guns with air compressors, etc. Manual spray guns are available in various configurations to suit a wide range of applications and processes. HVLP (high volume low pressure) gun offers the following advatages: the spraying is controllable, high transfer efficiency, can be used for a wide variety of materials, it is inexpensive to maintain because needs a reduced preparation and clean-up, is portable, the material consumption is reduced by 40%.

Their applications are: painting, food industry, pharmaceutical, dyestuff coating, oil, lubricating, rubber solution spray, textile spray, water based materials/paints, etc.

They are already applied on a large scale both by individuals and industrially.

#### 3.2.5. Surface Immobilization

Surface immobilization of the bioactive compounds was performed for a wide range of inert hydrophobic packaging polymeric materials. Surface modification of polymers can be performed either by physical, chemical (wet) or biological methods. Physical methods are preferred over chemical techniques because they offer greater precision, ease of process control and environment friendliness. Classical physical methods for modifying polymer surfaces include flame and corona treatment, ultraviolet light, gamma-ray, ion-beam techniques, low-pressure plasma and laser treatment. Physical methods either modify the surface layer or an extraneous layer is deposited on the top of the existing material—*coating*. The main advantage of plasma treatments is that the modification is restricted to the uppermost layers of the substrate, thus not affecting the overall desirable bulk properties. To achieve approximately similar surface modification, gamma irradiation should apply only in mild controlled conditions [146,147]. 

The physical methods are widely used due to their easier industrial scalability, no liquid reagents of any kind are needed in their application, thus is avoided the accumulation and handling of harmful waste [148]. The main effect of the surface activation methods is the formation of reactive species on the polymer surface like oxygenated groups as carbonyl, hydroxyl and carboxylic acid groups or nitrogen containing groups depending on discharge gas used in plasma device or after air exposure. On activated polymer surface bioactive compound can be either covalent or non-covalent immobilized Figure 2. Non-covalent way is based on electrostatic interactions when substrate and bioactive compound show opposite net charges, while ligand–receptor interactions, like in the case of biotin–avidin [149] are specific for affinity immobilization.

*Covalent attachment*/*conjugation* of bioactive compounds to functionalized polymer surfaces is realized by: hydrophilic, bifunctional, and/or branched spacer molecules. The main advantage of covalent immobilization is high stability of the formed layer and the active compound does not migrate into the packaged food product to affect its quality and safety. Active agents can be covalently linked directly to the polymer surface or by use of a spacer or crosslinker, which either share a permanent covalent bond between the polymeric substrate and the bioactive compound, or promotes covalent bond formation between the activated substrate and the bioactive compound without forming part of that link (“zero-length” crosslinkers). It can also perform optimization of the surface functionalization by using a polyfunctional agent, increased number of functional groups, tethering a bioactive compound to a solid substrate via a spacer molecule, covalently attach a bioactive compound to the functionalized polymer surface via an intermediary. The immobilization technologies are potential versatile as once functional groups are introduced to the polymer surface, a range of bioactive agents (e.g., enzymes, peptides) can be immobilized through standard chemical or bioconjugation techniques [150]. 

*Plasma and gamma rays assisted nanocoating and immobilization*. Plasma treatment and gamma irradiation are energetic processing methods that create reactive spots to which either oxygen- or nitrogen containing groups can be implemented on surface and functional groups and radicals are created depending on the type of discharge gas/atmosphere used inside reactors. The energy levels of entities generated in cold plasma are comparable to the bond energies of organic compounds and thus can facilitate surface functionalization reactions. The extent of functionalization can be controlled by selecting the discharge parameters [139]. On polymer surfaces three main effects can be obtained depending on the treatment conditions: a cleaning effect, an increase of microroughness surface and functionalization. According to the literature data acidic surfaces are obtained by O_2_ and air plasma activation of inert surface of synthetic polymers [151] and amphoteric surfaces are obtained by N_2_ plasma activation [152,153], in all cases after exposure to air for the same period of time of about 1 min. Nitrogen containing functionalities originate from plasma treatment itself and also, very likely, from the post-reaction with the venting gas. The oxygen atoms are both in the form of alcohol (C–OH) or/and carbonyl (C=O) groups, epoxy groups and radicals, while the nitrogen atoms appear as amino (C–NH_2_) groups. In order to develop new multifunctional active polymeric surfaces with special properties for food packaging applications based on inert polymer such as *polyolefins or polylactic acid and polyalkanoates*, the following solutions have been applied:

- Activation of polymeric substrates by non-solvent, environment friendly methods by using of gamma-ionizing radiation or cold plasma gas discharge.
- Stable layers have been deposited onto activated polymeric substrates using different coupling agents for covalent linking of active/bioactive formulations.
- Selected bioactive compounds were: chitosan/chitin, lactoferrin, vitamin E, natural vegetable oils with high content of antioxidant compounds as phenols or flavonoids mixtures.

Covalent grafting of bioactive compounds onto inert polymeric surfaces occurs by a two-step procedure:

Step I. *Polymeric surface activation*.

Surface activation techniques are applied in order to introduce the desired type and quantity of reactive functional groups which are able then to covalently attach a bioactive compound. The polymeric films/papers were exposed both to corona and to high-frequency plasma (air or nitrogen were used as discharge gas). The most important feature of irradiated polymers is the possibility of grafting for certain structures, because the radical sites are available for coupling. γ-exposure of polymeric films/papers was carried out in an irradiation machine at various irradiation doses from 2–30 kGy absorbed in air, at room temperature. Surface functionalization because of implementation of polar groups and radicals onto substrate surface provides desired changes in physical properties of the substrate surface (e.g., wettability, improved adhesion and biocompatibility, interaction with surrounding media) [154]. Although physical adsorption may be useful in some applications, covalent immobilization provides the most stable controllable bonds, between the bioactive compound and the functionalized polymer surface [155]. Optima conditions of activation by each method or compound to be linked were established by varying the exposure parameters and determination of the dependence of the surface wettability and morphology on these [146,147,156,157,158,159,160,161,162].

Step II. *Covalent bonding/grafting of bioactive compound onto activated polymeric surfaces*. 

After the corona, plasma discharge or γ-irradiation pre-treatment, the polymer surface was enriched with oxygen-containing groups, such as carboxyl, carbonyl, hydroxyl, ester groups and/or nitrogen containing groups. The covalent attachment between a polymeric surface and an active compound is achieved by formation of amide, ether, ester and thioester bonds, created between the hydroxyl, amine, imine, carboxylic acid and thiol groups of the active compounds of interest which may possess intrinsically (or are incorporated in their structure) and the functional groups created on the substrate [156,157,163,164]. After cold plasma/γ-ray exposure the polymeric films/fibres/papers were removed from the treatment chamber and then immersed into the bioactive compound solution (chitosan (CHT), grape seed oil, clove oil, etc.). The dipping/immersion, spreading or electrospinning/electrospraying of such solution onto activated surface lead to coat of the activated surface. For covalent bonding of CHT or other bioactive compounds onto activated surfaces, the Ethyl-3-[3-dimethylaminopropyl] carbodiimide hydrochloride) (EDC), -*N*-hydroxysuccinimide (NHS) or 1′-Carbonyldiimidazole (CDI) coupling agents were used.

Comparing various methods of chitosan deposition onto cold plasma and γ-irradiation activated PE surface it was established that for the same concentration of the chitosan solution the most efficient method was the immersion in respect with homogeneity of surface and thickness of deposited layer but the electrospraying appeared to be more versatile method, because it allows a more precise control of the chitosan content deposited onto the surface by varying the deposition time. 

Attenuated Total Reflectance Fourier TransformIinfrared spectroscopy (ATR-FTIR), X-ray photoelectron spectroscopy (XPS) and potentiometric titration proved CHT presence on the PE surface and also new bonds are evidenced mainly amide groups or ester groups because of interaction of –NH_2_ or –OH groups of CHT with carboxyl group implemented on PE or PLA surface by radiation activation of surface. Other reactions were also possible because of the high reactivity of the active species created by plasma exposure/gamma irradiation and the functional groups of chitosan [157,165] Chitosan coating improved the oxygen barrier properties of PE (Oxygen Transmission Rate (OTR) of PE decreased 3 times being of 1066 mL/(m^2^·day) while OTR of the uncoated PE is 3833.36 mL/(m^2^·day). The potentiometric and polyelectrolyte titrations, zeta potential determination showed that some amount of chitosan desorbed faster from the surface until equilibrium was reached and also that the grafted chitosan layer was more stable than the physically adsorbed one. In the case of grafting, a thin chitosan layer was irreversibly immobilized on the PE surface. The obtained materials combine the antibacterial, antifungal, bioadhesivity, biocompatibility, biodegradability of chitosan [146,150,156,157,163,164,165,166,167]. with the biological functions and antioxidative activity of vitamin E. Vitamin E is also known for its activity to prevent cell membrane damage by inhibiting peroxidation of membrane phospholipids and disrupting free radical chain reactions induced by formation of lipid peroxides [168]. 

**Figure 3:**
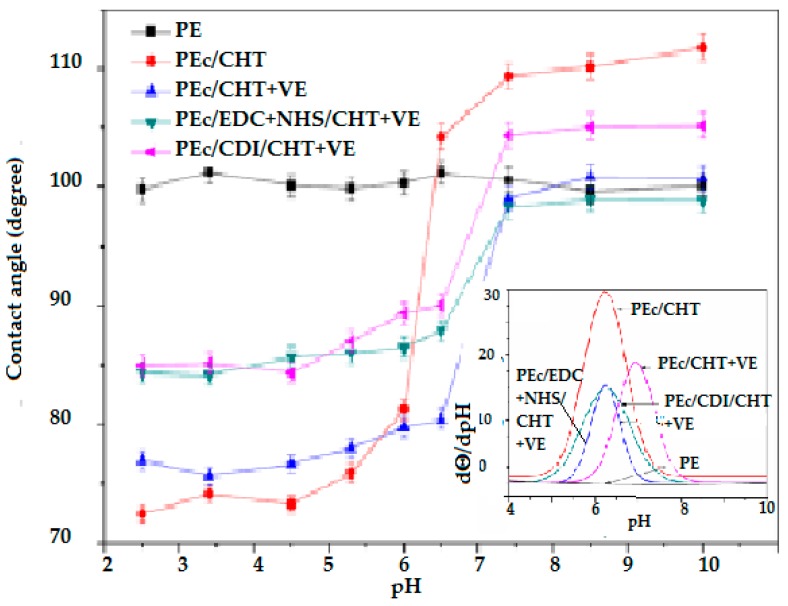
Contact angle (θ) versus pH titration curves and derivative curves of dθ/dpH (insert) for chitosan/vitamin E-coated PE films adapted from [157].

The pH responsiveness was evidenced was evidenced by contact angle measurements using buffered solutions with pH varying in the 2–11 range and it appears by sudden switching from hydrophilic to hydrophobic surface at critical pH ≈ 6. The contact angle of the polyethylene surface remains constant over the entire studied pH range (Figure 3). 

Also, vegetable oils (clove oil, argan oil, thyme oil, tee tree oil and so forth) and combinations chitosan/vegetable oils have been tested to combine antimicrobial activity of CHT with those of vegetable oils and their antioxidant, biological function and nutrition [169]. They were covalently immobilized both on the activated substrate of PE and PLA and the results indicated their synergism in preventing microbial growth on fresh crude cheese, beef meat, poultry minced meat and fresh apple juice [136,140,146]. Both plasma activation and γ-irradiation have great influence on the antimicrobial activity being known that they are relatively simple and quite safe microbial sterilization techniques that are utilized in a variety of applications for their low operating costs and non-polluting capabilities. In the case of lactoferrin immobilization it was established that gamma irradiated samples showed higher activities than those nitrogen plasma activated [150]. 

Lignocellulosic materials usually display a very low microbial resistance and microbial contaminations might be an additional issue to be taken into account. Conferring antibacterial activity to lignocellulose-based products may represent a main functional property which is useful not only for advanced food packaging [113] but also for textile applications. This can be realized by incorporation of aldehydes, epoxy, carboxylic acids and so forth [160,170] followed by attachment of some phenolic structures which can react at different extents with plasma/γ-irradiated activated surfaces and therefore they will be grafted on the lignocellulosic material surface to develop covalently bound antimicrobial products. The cellulose/chitin mixture and kraft paper were modified using different types of plasma: air, oxygen, nitrogen and argon activation followed by reaction with various phenolic compounds such as p a-hydroxybenzoic cid (HBA), galic acid (GA) eugenol (Eu) and grape seed oil (GO) and rosehip seed oil (RO) and it was found efficient for inhibiting growth of microorganisms onto fresh crude cheese and beef meat. [160,171]. The antimicrobial activity was increased up to 100%. The rosehip seeds oil imparted the best antimicrobial properties to cellulose/chitin mix substrate. As functionalizable substrate is a biodegradable polymer and surface treatment was realized by physical methods of activation (cold plasma and gamma irradiation in mild conditions), the obtaining of the materials with desired surface properties avoided the harmful environmental concerns.

#### 3.2.6. Wet Methods

Wet Methods involve both corrosive liquids to which the polymer substrates are directly exposed, like piranha solution (dissolved hydrogen peroxide and sulfuric acid), combined sodium hydroxide and sulfuric acid, chromic acid, potassium permanganate and nitric acid [172] and graft copolymerization. The first method has not a wide applicability but graft copolymerization received a wide scientific and applicability interest. 

#### 3.2.7. Photografting

Photografting method is a free radicals photografting procedure which occurs onto a polymer surface exposed to UV light in the 315–400 nm range in the presence of photoinitiators (e.g., benzophenone, anthraquinone, thioxantone, phenyl azide, polymers, curcumin) and monomers which usually bear ketone groups [173]. The active agent can be directly incorporated during photografting, or by its subsequent immobilization after grafting of a polymer chain with reactive functional groups (e.g., acrylic acid).

#### 3.2.8. Biological Methods 

Biological methods consist in: pre-adsorption of proteins, drug, enzyme immobilization, cell seeding and pre-clotting [174]. 

#### 3.2.9. Chemical Vapour Deposition (CVD)

In typical *CVD*, the substrate is exposed to one or more volatile precursors, which react and/or decompose on the substrate surface to produce the desired deposit [175]. Diamond-like carbon (DLC) has been investigated as coating materials for industrial applications due to its outstanding characteristics such as high hardness, wear resistance, chemical inertness and biological compatibility. The demand for polymeric materials has also been increasing and highly functionalized polymers are much desired for wider applications to the production materials ranging from biomaterials to eco-friendly materials. In order to modify the polymers for highly-functionalized and highly biocompatible materials, DLC can be effectively nanocoated on polymeric materials, especially on polymeric surfaces through CVD methods. By nanocoating such DLC, a new type of polymer/DLC nanocomposite could be established for the use in biomedical devices and food packaging. 

#### 3.2.10. Atomic Layer Deposition (ALD) Method

VTT Technical Research Centre of Finland developed a fully recyclable nanocoating for food and pharmaceuticals [176] which is thin, light and air-tight, conformal and pinhole-free coating which follows the contours of the coated material, with excellent gas permeation resistance for packaging materials by ALD method. These thin bio-based packaging materials have gas permeability properties similar to those of existing dry food packages and pharmaceutical blister packs protecting the products from humidity, drying or oxidation. ALD coating allows integrating different functions into the packaging material, such as prevent water, oxygen, humidity, fats and aromas from permeating the packaging and protect the surface from stains and bacterial growth. The ALD technology improves the humidity tolerance and performance of bio-polymers, reducing the need for oil-based plastics.

#### 3.2.11. Layer-by-Layer Assembly

By layer-by-layer assembly deposition can prepare active packaging coatings by the incorporation of active agents either between layers or within the structure of an individual polyelectrolyte [177]. Onto a polymeric activated substrate can be deposited by mutual attraction of polyelectrolytes with opposite charges or even by covalently attached of a polyelectrolyte such as proteins, polysaccharides, synthetic polymers of successive alternate layers [135,178]. Deposition can be accomplished either by submersion of the substrate into polyelectrolyte solutions or by spraying of solutions onto the substrate. The depositions are the presence optimized by adjusting the pH of their solutions for a full protonation or deprotonation, by maximizing of charge [179]. Between layers can be either only electrostatic interactions [180], and/or covalent bonds through the use of crosslinkers. The number of deposited layers is limited by a saturation state characterized by a thickness and stability of the system [181,182]. Jokar et al. produced coatings by the layer-by-layer deposition method. LDPE films were ultrasonically washed with acetone, functionalised in dilute aqueous solution of polyethylenimine for 10 min and sequentially dipped into anionic silver colloid dispersions containing PEG-capped silver NPs or cationic chitosan for 10 min. Silver particles were 18–32 nm and 22–30 nm in size in melt-blended and coated composites, respectively and their migration in simulated media as water, 10% ethanol, 3% acetic acid and apple juice depends on silver concentration and temperature [183]. Covalently bonded multi-layered films were assembled also using the LBL techniques by using polymers bearing functional groups capable of reacting with one another or with a bi-functional agent (diamines, diimides or dialdehydes). Covalent cross-linking increased the modulus and stability of the films. 

#### 3.2.12. Ultrasonic Nozzle Systems for Nanotechnology Coating Applications

Sono-Tek ultrasonic nozzles are suitable to depositing precision nanotechnology coatings for spray applications in research as well as production volume spray processes [184]. Uniform nanolayers of solutions onto any size or width substrate (including moving webs of material) are deposited and nano-suspensions are homogenously dispersed the onto a substrate, including suspensions that tend to agglomerate easily which are continuously break due to the mechanical vibration of the nozzle which is much efficient than air spraying. Sono-Tek systems employ the ultrasonic nozzle technology which enables to be easily scaled up from R&D to high volume production.

#### 3.2.13. Plasma Nano-Coating of Beverage Cans

Interior and possibly even exterior coating of the aluminium cans and tubes is required in the food industry because the direct contact between the product and the packaging can lead to aluminium corrosion and the food could spoil. For example, the pasteurization increases corrosive impact on packaging. PlasmaPlus^®^ coating technique uses the plasma jets deposit micro-fine glass-like nano-coats which form a highly effective protective film on the packaging with minimum material usage. Plasma coats provide a perfect quality imprinting [185]. Nobile et al. [186] produced the coating in polyethylene oxide on the surface of PE film by plasma-based vapour deposition. Transmission electron microscopy (TEM) imaging revealed AgNPs about 90 nm in size deposited on the films. The migration of silver ions from nanosilver coatings onto PE films studied by immersion in distilled water, acidified malt extract broth and apple juice at 44 °C for 5 days. It was found that the silver content in the solutions was the highest in the distilled water simulant (1–1.9 mg/kg), with lower concentrations in the malt extract broth and apple juice (0.2–0.38 mg/kg). 

#### 3.2.14. Electrospinning/Electrospraying

**Figure 4:**
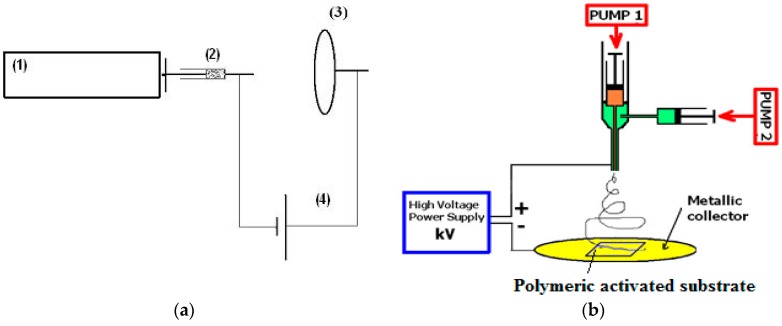
Representative experimental set-up of the uniaxial (**a**): (1) pump; (2) syringe; (3) collector; (4) high direct voltage (0 to 30 kV) power supply; or coaxial (**b**) electrospinning device used for immobilization of vegetable oil into chitosan and bioactive layer deposition onto polymeric activated substrate adapted from [146]; Vasile et al. (2016).

Electrospinning/electrospraying has recently gained much attention because of its versatility in processing a wide range of polymer and biopolymer materials. It has the ability to produce fibre/particle diameters within the submicron and nano range using electric field which cannot be achieved by using conventional-generating technologies. Nanofibers have wide applications in industry such as food packaging coatings, composite materials, carbon nano tubes, inorganic fibres and tissue scaffolds, filtration, medical, membranes and so forth [187]. Electrospinning has been used to create a well-dispersed “concentrate” or master batch that will act as a carrier of well dispersed nanoparticles. It is an electro-hydrodynamic process, comprising electrospraying and electrospinning techniques which produces nanostructured fibre-based and particle-based materials both in laboratory and industrial scale. The devices used for the mono-axial and co-axial electrospinning/electrospraying method is presented in Figure 4.

*In monoaxial electrospinning* the device consists of a direct current high voltage supply, a metal plate collector sustaining the polymeric substrate, a pump and a syringe oriented perpendicularly to the metal plate with the low diameter needle (Figure 4a). A high direct voltage (0 to 30 kV) between the metal plate and the syringe needle is applied. The polymer solution is extruded through the needle tip by the syringe pump at a constant flow rate. At the point of ejection (the needle tip), is created a polymer jet because of the electric charge repulsion outgoing the solution surface tension. Based on electrostatic field between nozzle (needle tip) and collector, the dry polymer or solution are collected on the surface of collector screen. On the collector a very thin polymer layer is deposited, whose morphology as nanofibres or nanoparticles, depends on rheological properties of the solution [156] and composition of electrospinnable solutions [157,188]. This device has some disadvantages such as drying which can be controlled by the distance between nozzle and collector.

*In co-axial electrospinning* two syringes are used one of them being provided with a co-axial needle oriented with the needle perpendicular to the metal plate. It offers the possibility of depositing a bioactive layer/coating containing two bioactive compounds onto activated surface achieving the encapsulation of less stable or liquid compound (as vegetable oils) into a polymeric one so core-shell morphology is obtain which can increase the coating functionality and protection of the labile bioactive component and also the use of nanomaterials in other technology. Encapsulation of the vegetable oils in chitosan was achieved by coaxial electrospinning method and immediately a coating of the nanoparticles/nanofibres was deposited onto polymeric substrate [146,189]. This technique may also be used with one of the two syringes with the polymeric solution and the other one with the same solvent able to dissolve the polymeric matrix. This approach is to improve the processability, being particularly interesting to scale up the electrospinning technique from lab scale to the industrial sector.

Electrospun fibres have a very high specific surface and porosity and they are able to encapsulate active substances. A wide range of applications are known for such fibres as improvement of physico-chemical and functional properties of biopolymer based materials by means of a controlled release of active compounds or by enhancing the dispersion of nano-additives into the biopolymer matrices. The nano encapsulation/ entrapment can reduce the amount of active ingredients needed to obtain certain functionality and also their controlled release [158,188,190]. Nanofiber-based systems for antimicrobial food packaging and food contact surface applications contain antimicrobial fibre-base mats as coating of the packaging material, improve their activity in maintaining an optimal effect during the food storage period. Furthermore, the high surface to volume ratio of electrospun mats offer an efficient and prolonged delivery of loaded bioactive compounds. 

An antimicrobial active multilayer system was obtained from a commercial polyhydroxyalkanoate substrate (PHA) and an electrospun PHA coating with in situ-stabilized silver nanoparticles (AgNPs) with satisfactory thermal, mechanical, barrier and antibacterial properties. It reduced the bacterial population of *Salmonella enterica* below the detection limits at very low silver loading of 0.002 ± 0.0005 wt %. Also, polylactic acid (PLA)/Silver-NP/Vitamin E bionanocomposite has been prepared by electrospinning and they showed a good antimicrobial activity against both gram positive and gram-negative bacteria [190]. As a result, this method provides an innovative route to generate fully renewable and biodegradable antimicrobial materials for food packages and food contact surfaces [191]. The choice of the manufacturing process depends on specific application requirements of the coating. As an example, the percent reduction against the fungus *A. Níger* after 14 days, for the three multilayer samples coated with AgNP/polyethylene nanocomposites with the silver nanocomposite layer of AgNO_3_ suspension (black) all at 1.0 wt % of silver content depends on the coating method, the most efficient being spraying one which gave a reduction of more than 70% while laminated and casting method reduction were of 20% and 45%, respectively [192].

## 4. Applications

Nanocomposites and nanocoatings applications in food packaging depend both on the properties of matrices/substrates and activity of ingredients. Nanoclay composites (usually montmorillonites) are used as flexible and rigid food packaging because of their excellent barrier characteristics in packaging of processed meats, cheese, confectionery, cereals and boil-in-the bag foods, items obtained by extrusion-coating process together with the paperboard are useful for fruit juice and dairy products and the beer and carbonated drinks bottles are manufactured by co-extrusion processes. For example, the PET bottles contain nanocore and nylon beer bottles with nanoclays show improved barrier characteristics. The active agents functions include antimicrobial, antifungal, antioxidant, biocatalyst, external stimuli responsiveness, additives that absorb undesirable component from food such as ethylene, moisture and odour and so forth They are incorporated into matrix or coating to enhance the quality and shelf life of the food and are released in a controllable manner, the procedure being much efficient then direct addition into the food because they may react with other food components and loss their activity or affect food sensorial characteristics.

There are many formulations including such antimicrobials, antioxidants and biocatalysts, some of them being summarized in Table 3 and Table 4.

### 4.1. Antimicrobial/Antibacterial

Nanomaterials are more and more used to target bacteria in textile industry, marine transport, medicine and food packaging as antibacterial coatings and other materials [253]. Antimicrobial packaging role is to control the growth of pathogenic and/or spoilage microorganisms in packaged products. The diminishing and control bacterial colonization by the modification of the surfaces can be achieved [254]. As antimicrobial agents, essential oils, organic acids, peptides, enzymes and biopolymers are applied and nanoparticles like AgNP, MgO, CuO, Cu, ZnO, Cd selenide/telluride, chitosan, carbon nanotubes are used. Antimicrobial plant extracts from many plants and fruits such as thyme, clove and tea tree, rosemary oil or powder [169], sea buckthorn (*Hippophaë rhamnoides* L.) leaves and inner bark of pine trees (*Pinus silvestris*) [255] used in food packaging provide a healthy alternative. They contain aromatic and phenolic compounds that are responsible for their antibacterial properties and antioxidant activities. They can be applied as components in chitosan films or nanofibres obtained by electrospinning deposited on PLA [256]. Such essential or cold press oils exhibited selective antibacterial and/or antifungal effect both as a solvent extract and as a coating against three food spoilage fungi—*Fusarium graminearum*, *Penicillium corylophilum* and *Aspergillus brasiliensis*—and three potential pathogenic food bacteria as *Staphylococcus aureus*, *Escherichia coli* and *Listeria monocytogenes* and also against *Pseudomonas aeruginosa.*

TiO_2_ and ZnO are non-toxic and approved by FDA as GRAS. TiO_2_ nanoparticles exhibiting bactericidal and fungicidal effect against *Salmonella choleraesius*, *Vibrio parahaemoliticus*, *Staphylococus aureus*, *Diaporthe actinide* and *Penicilinum expansum.* It was ultrasonically dispersed ethylene-vinyl alcohol copolymer films to obtain active packaging. It showed excellent bactericidal property under UV light [257]. Because of their very small dimensions, the nanomaterials are able to attach many biological molecules, therefore showing a greater efficiency [258]. ZnO is efficient both against Gram-positive and Gram-negative bacteria. NanoZnO coated films exhibit antimicrobial effects against *L. Monocytes* and *S. Enteritis*.

Have been proposed several mechanisms for the antimicrobial activity of nanoparticles as: directly interactions with microbial cells and interruption of the trans-membrane electron transfer, disrupting/penetrating the cell envelope, oxidizing cell components or producing secondary products as reactive oxygen species (ROS) or dissolved heavy metal ions. Secondary processes also can occur. For example, the antibacterial activity of silver ion is reduced by the protein rich food because it can bind the cysteine, methionine, lysine and arginine [259]. Nanosilver antimicrobial activity is explained by adhesion to the cell surface, degrading lipopolysaccharides and damaging the membranes, largely increasing permeability, by penetration inside bacterial cell, damaging DNA [260] and releasing antimicrobial Ag^+^ ions by AgNPs dissolution which then bind to electron donor groups in biological molecules containing sulphur, oxygen or nitrogen [261]. Silver ions can be released to destroy food spoilage. They show also a high temperature stability and low volatility. Nanosilver is useful as coatings component where the antimicrobial action occurs at the surface as on containers, mugs, dishes, cutlery, fridges, chopping boards and so forth [262]. It was found effective against numerous species of bacteria including *E. Coli*, *Enterococcus faecalis*, *Staphylococcus aureus* and *Epidermidis*, *Vibrio cholera*, *Pseudomonas aeruginosa* and *putida* and *fluorescens* and *oleovorans*, *Shigella flexneri*, *Bacillus anthracis* and *subtilisi* and *Cereus*, *Proteus mirabilis*, *Salmonella enterica* typhmurium, *Micrococcus luteus*, *Listeria monocytogenes* and *Klebsiella pneumoniae* [1,263] some of them presenting resistance to other potent chemical antimicrobials. Migration studies for nanosilver containing PE composites or coatings [264] in different simulants as malt broth, apple juice, distilled water and 10% ethanol showed good results.

The NanoBioMatters highly efficient antimicrobial product and BactiBlock^®^ technology are based on silver-functionalized nanoclay. MgO and ZnO nanoparticles could provide cheap, safe alternative to expensive nano-sized silver as a heavy metal not suitable for human contact. The combinations of α−tocopherol with nisin or chitosan as coating or biocomposites confer both antimicrobial and antioxidative properties [136,140,225]. 

Antimicrobial activity of the chitosan and engineered nanoparticles is explained by several mechanisms including interactions between positively charged chitosan and negatively charged cell membranes, increased membrane permeability and rupture and leakage of intracellular material. They are ineffective at pH values above 6 because of the absence of protonated amino groups [265]. Another two antimicrobial mechanisms as chelation of trace metals by chitosan, inhibiting enzyme activities and, in fungal cells, penetration through the cell wall and membranes to bind DNA and inhibit RNA synthesis were also proposed [167].

*Carbon nanotubes* also have antibacterial properties. They are efficient against *E. coli*, possibly because puncture microbial cells [266] but is possible to be cytotoxic to human cells, to skin and lungs in processing stages rather than for consumers, therefore their migration to food must be carefully controlled.

### 4.2. Antioxidant

Oxidative degradation of food products happens during transport and storage producing lipid rancidity, colour loss (e.g., oxidation of carotenoids, chlorophyll, anthocyanins) and vitamin degradation. The antioxidants (e.g., free radical scavengers, metal chelators, singlet oxygen quenchers, oxygen scavengers) are commonly incorporated in food packaging by blending with polymers during processing (mixing, extrusion) [267,268]. The antioxidant active packaging technologies include oxygen scavengers, manufactured as sachets or labels and by the development of migratory and non-migratory antioxidant coatings. In the first case is possible a controlled release of an antioxidant (migration is 20 times less than the legal limits for the European Union) and sometimes does not requiring direct contact with the food [269]. Non-migratory antioxidant coatings may also be applied by covalent immobilization by means of the functional groups on the surface of packaging materials and shows advantages that do not alter sensorial properties of the packaged food product [270].

### 4.3. Biocatalytic

Enzymes onto solid support materials provide biocatalytic coatings for active packaging by catalysing some reactions. Biocatalysts are used in ingredients production and breakdown of undesirable components which are harmful or may decrease product quality. Biocatalysts are very sensitive, therefore the immobilization method onto and into solid supports must preserve their thermostability, optima pH and solvent stability and usually so-called “in-package processing” is applied to avoid changes during processing, transport and storage. Antimicrobial enzymes (e.g., lysozyme) are incorporated into active packaging coatings via blending, non-covalent binding for controlled release and covalent immobilization [150,271,272]. Their compatibility with matrix can be achieved by surface functionalization and cross-linking techniques [273]. The thermostability and pH stability of the bound enzyme determines the success of the coating method.

### 4.4. Barrier Applications of Polymer Nanocomposites

A critical issue in food packaging is that of migration and permeability [170,274] to atmospheric gasses, water vapour, or substances contained within both in the food being packaged or even the packaging material itself. As examples, in packages for fresh fruits and vegetables high barriers to migration or gas diffusion are undesirable because their shelf-life depends on oxygen for sustained cellular respiration while the plastics for carbonated beverage containers must have high oxygen and carbon dioxide barriers to prevent oxidation and decarbonation of the beverage contents. Though polymers possess numerous advantages their major drawback is an inherent permeability to gasses and other small molecules. PET provides a good barrier to oxygen (6–8 nmol/ms GPa) while this is very low for HDPE (200–400 nmol/ms GPa). The situation is opposite in respect with barrier against water vapour [170]. Oxygen and carbon dioxide barriers are necessary for plastics used for carbonated beverage containers. EVOH exhibits excellent oxygen transmission rate (OTR) values under dry conditions but under very humid conditions (relative humidity > 75%) it can possess OTR values more than an order of magnitude higher due its swelling and plasticization in the presence of diffused water molecules [275]. Bio-derived polysaccharide (starch, chitosan, pullulan) based packaging show even larger dependence of their OTR on humidity level, which has severely limited their usefulness. Thermoplastic biopolymers like PLA or polycarprolactones (PCLs) have good tolerance to moisture [276]. Because of the tortuosity effect created by the presence of highly crystalline CNCs (1% CNC) in the PLA-based nanocomposites, which increased degree of crystallinity all barrier properties are significantly improved (WVP (lower ∼ 40%; OP ∼75% than neat PLA films). PLA/CNC with 1% nanofiber showed also improved of elongation at break [277,278,279]. Because of this specific dependence, complex multilayer films (by co-extrusion) [280] or polymer blends and composites are often preferred [281]. A high oxygen barrier, water sensitive material like EVOH was sandwiched between two layers composed relatively hydrophobic polyethylene [172]. Multilayer films and polymer blending require the use of additional additives and adhesives that complicate their regulation by federal agencies and arise recycling difficulties. Polymer nanocomposites (PNCs) should solve most of the above-mentioned problems.

### 4.5. Stimuli Responsive Nanocomposites/Nanocoatings

Stimuli responsive nanocomposites/nanocoatings prepared using “stimuli-responsive, “smart,” “intelligent” or “environmentally sensitive” polymers [282,283,284,285]. These polymers respond to environmental changes by changing their conformation, solubility, hydrophilic/hydrophobic balance, reaction rate, swelling and release behaviour (for hydrogels) and molecular recognition. Their response to the external changes are reversible, “switching” may occur repeatedly and occurs in a narrow interval of stimuli variation. Responsive polymers have found applications as free chains dissolved in aqueous solutions, as covalently or noncovalently crosslinked hydrogels and immobilized adsorbed or surface–grafted onto solid surfaces [286]. Surfaces modified with stimuli-responsive polymers dynamically modify their physico-chemical properties in response to changes in their environmental conditions. Stimuli-responsive surfaces have been created by the immobilization of reversible thermally responsive elastin-like polypeptide, on a glass surface [287]. The distance-dependent colorimetric properties of gold nanoparticles, enable to determine the effect of different variables on the lower critical solution temperature (LCST) at the solid–water interface. A poly(ethyleneimine)–poly(dimethylsiloxane) mixed brush switched spontaneously from the hydrophilic state in water to the hydrophobic one in air, therefore materials show a poor adhesion in a changeable environment. The surface coatings fabricated from mixed poly (ethylene oxide) (PEO)–poly(dimethylsiloxane) brushes or from fluorinated nanoparticles were adaptive to liquid and vapour environments so that the surfaces were spontaneously transformed to non-sticky states in air and in water. Smart and self-healing coatings have been also prepared. Colloidal particles obtained by the emulsion copolymerization of acrylate and fluorinated acrylate monomers form stratified film morphologies in superhydrophobic surfaces [288]. Thermoresponsive poly(*N*-isopropylacrylamide)-based microgels and assemblies have a diverse range of applications for example, biosensing, smart coatings and so forth [289]. 

*Sensory Packaging* is a category of devices used in intelligent packaging as nanosensors to monitor and report on the condition of food, communicate the degradation of product or microbial contamination and give information on history of storage, avoiding inaccurate expiration date increasing security and food safety. It helps food industry and retailers to now if the food package has been opened or tampered. This could be achieved by using a nanocrystalline indicator in form of an oxygen intelligence ink printable on most surfaces. This ink contains an UV light activated nanocrystalline particles of a semiconductor (usually TiO_2_) or nanophosphourus particles. These particles are white in daylight but will fluoresce when exposed to light of certain wavelengths. The colour pigments can be also used as micro-colour codes in packaging and labelling applications. The development of sensory packaging which monitor the conditions of pharmaceuticals and foods are those affected by changes in temperature, humidity and shock. Nanobarcodes developed by Nanoplex are written into fluorescent microspheres by photobleaching. Coating on PET bottles will expand the packaging of sensitive juice or beer beverages 

## 5. Possible Risks

Consumers are hesitant to buy nanotechnology foods or food with nanotechnology packaging. However, some results suggest that nanotechnology packaging is more beneficial than nanotechnology foods. The main concern with the application of the nanotechnology in food packaging is related to the fact that the very small sizes of the nanoparticles have different chemical and physical properties in respect of macroscale materials and it is possible they could cause health problems. New packaging materials must have good barrier properties to oxygen, carbon dioxide, water vapour and flavour compounds. This controlled release packaging is another example of a nanomaterial application in active packaging. Nanoclays can be used as carriers for the active agents with high efficacy because it is highly dispersed in the polymeric matrix and, hence, exposed more efficiently to the substance on which it is required to act. Potential implications for consumer safety are migration of nanoparticles. They can enter into body through ingestion, inhalation or dermal contact leading to health effects of exposure to some insoluble, persistent nanoparticles. Such health effects are currently not known. Nanoparticles also migrate to foodstuffs with possible adverse effects on food quality. Other concern is with degradability of bio-polymers and formation of degradation products with possible adverse effects. Also, may appear potential environmental impacts of nano-polymer composites and some problems with end-of-life treatments as recycling, re-use and disposal. Detecting the migration of nanomaterials requires more sensitive analytical techniques due to the complexity of the nanomaterials and because they represent only a very small portion of the bulk food. Migration into food can cause both undesirable organoleptic changes (migration of TiO_2_ into lipid matrix results in rancidity) and in some cases nanoparticles of bioactive compounds are intended to be released deliberately.

A considerable number of migration studies were found for nanosilver containing polymer composites or coatings. Overall the results from these studies suggest the production method of nanocomposites (e.g., incorporation or coating, surfactant modification), starting silver concentration, temperature, time and choice of contact medium are all factors which may have an effect on the extent of silver ion migration into food simulants. In general, the rate of migration increases when nanosilver is coated onto the food packaging material or surfactants are added, when the storage temperature and length of storage increases and the acidity of the contact medium increases. There appears to be a specific time of storage, after which a steady state release of silver is achieved. This is supported also by a repeat contact migration experiments, which found silver migration decreased considerably (by an order of magnitude) after first contact. Nevertheless, there is some evidence to suggest that if silver nanoparticles do migrate into food/food simulants, they would most likely dissolve quickly into ionic silver. The majority of the migration studies found for nanosilver food packaging composites have shown levels of migration of ionic silver into foods and food simulants below the European specific migration limit (SML) of 0.05 mg Ag/kg food, suggesting low consumer exposure and subsequently low risk of adverse effects. However, there are also several studies, in which migration exceeded this limit. This indicates that for new food packaging products containing nanosilver, the migration experiments should be conducted in each particular case. Migration of intact nanoparticles into food simulants is negligible, implying consumer exposure to these materials is likely low. This suggests there is low potential for safety issues related to the nanosize level of the materials incorporated into food packaging. If they migrate in nanoparticulate form, it would be anticipated at the resulting low concentration in food that many of the metal oxide nanoparticulates would likely dissolve into their ionic forms upon contact with acid foods or stomach acid. Theoretically, potential consumer exposure to nanomaterials incorporated into food packaging will appear if: they migrate into foodstuffs or drinks from the packaging, or if the nanocomposite polymers degrade and ‘dissolve’ into food or drinks. Migration can theoretically occur if nanoparticles desorb from the surface of the packaging material due to weak bonding at the surface (only really relevant for coatings), diffuse into foods as a result of a concentration gradient, or dissolve resulting in ions released into food [209,290]. However, currently, there are no internationally protocols or standards concerning nanomaterials characterization or to assess their implications on the consumers health [291,292,293] 

## 6. Commercial Level

The companies producing nanoreinforced food packaging materials are Color Matrix Corporation manufacturing Imperm a high barrier nylon multilayer bottles films; Mitsubishi Gas Chemical Comp. Nanocore producing NANO-N-MXD6 for PET bottles; Lanxess for Durethan KU2-2601; Honeywell polymer with Aegis NC and OX as coating PA6 on paperboard and coinjection PET bottles [33], NanoSealTM Barrier Coating and Bairicade XTTM Barrier Coating (from NanoPack Inc, Wayne, PA, USA) described as a water based coating comprised of a master batch and a liquid dispersion of clay platelets (‘nano’ or particle size is not mentioned). The coating is applied to traditional packaging films to enhance gas barrier properties and is stated to be approved for indirect food contact (i.e., used with dry and moderately dry food applications).

Nanocor Inc. Deveoped Imperm^®^ as multilayer PET bottle and sheets is used to improve barrier properties and Nylon MXD6 Nylon MXD6-Ultra Barrier System [294], Duretham^®^ KU 2-2601 (LanXess GmbH, Cologne, Germany) nylon nanocomposites for films and paper coating designed for medium barrier applications, requiring excellent clarity; Aegis^®^ (Honeywell, NJ, USA) a polymerized nanocomposite film incorporating an active oxygen scavengers and passive nanocomposite clay particles.

“Micro-pore-plugging” film as single nanolayer on PET, manufactured by Tera-Barrier, provides transparency and stretchability for applications in food, medical and other packaging markets. Coating of 400 nm is obtained by slot die and has WVTR (40 °C and 100% RH) of 5 × 10^−2^ g/(m^2^ day), transparency 86% (PET 87%) at 550 nm, stretchability > 5%, average roughness 10.3 nm. Sidel trade name used for less sensitive foods are a coating of about 200 nm thick obtained inside PET bottle wall by acetylene plasma. 

Barrier films are manufactured by Clairiant and Nanocore from PP and respectively Nylon with organoclay for packaging or PET beer bottle. 

Purdue University developed a large-scale manufacturing process that may change the way some grocery store foods are packaged. This new manufacturing process uses cellulose nanocrystals as advanced barrier coatings for food packaging. The Purdue manufacturing technique is a roll-to-roll manufacturing process using waterborne polymer systems being scalable [77]. By their studies it appreciated that food packaging is a growing billion-dollar market and overall predicted growth is expected to reach 6 percent by 2024. Advanced barrier coatings, which help to protect grocery items such as foods and beverages, are growing by as much as 45 percent each year. 

## 7. Conclusion and Future Trends

The global trade of active food packaging was estimated to be around US $12,000,000,000.00 in 2017 [295]. The active food packaging market is dominated by oxygen scavenging and moisture absorption applications [296,297]. Consideration must also be given to requirements of different regulatory agencies, under the jurisdiction of the Food and Drug Administration (in the United States), the European Food Safety Authority (in the European Union), or for each country. Non-migratory technologies produced by either covalent immobilization, cross-linked layer-by-layer deposition, or some photografted coatings offer a potential regulatory benefit. Migration testing using standardized simulants (water, 3% acetic acid, 15% ethanol, olive oil, iso-octane and 95% ethanol) must be performed to quantify levels of migrants in packaged product systems [298]. A benefit to coatings over bulk material modification is that bulk material properties should remain intact. However, the influence of the coating on processability, thermomechanical properties, barrier properties and seal strength must be characterized [299]. Rigorous application tests must also be performed to ensure that neither material conversion steps nor end use result in delamination of active coatings. Many of the coatings technologies have potential for scalability to roll-to-roll, high throughput coating operations. Finally, while incorporation of active agents and specialized packaging processes will indeed increase material cost, the opportunities for new products, enhanced safety and reduced waste of packaged goods highlight the potential for increasing product value through smart integration of active biodegradable packaging coatings.

Nanotechnology has the potential to generate new food products and new food packaging. Analyses of individual data showed that the importance of naturalness in food products and trust were significant factors influencing the perceived risk and the perceived benefit of nanotechnology foods and nanotechnology food packaging [300,301,302,303]. 

Nanotechnology has been found to be a promising technology for the food packaging industry. It has proven capabilities that are valuable in packaging foods, including improved barriers; mechanical, thermal and biodegradable properties; and applications in active and intelligent food packaging including anti-microbial agents and nanosensors, respectively. However, the use of nanocomposites in food packaging might be challenging due to the reduced particle size of nanomaterials and the fact that the chemical and physical characteristics of such tiny materials may be quite different from those of their macro-scale counterparts. Migration studies must be conducted to determine the amounts of nanomaterials released into the food matrices [292]. Nanoscale dimensions can increase significantly the physical interactions, physico-chemical and chemical interfaces in materials [304]. The morphologies obtained for the nanocomposites and the ability to modify the interfaces are essential to maximize the properties. Surface treatment and intensive in optima conditions mixing are key solutions determining the nanomaterials performance. The combinations between nanofillers/matrix and bioactive compounds allow wide possibilities of mechanical, thermal, optical, electrical, barrier properties and multifunctionality to create good food packaging materials. Coating technology has undergone a wide variety of changes in the last few years to become a flexible coating process for innovative surface functions. Today, barriers and multifunctional surfaces can meet complex requirements for a wide range of applications in a variety of sectors. New technological solutions for upgrading smart products as other emerging trends are developing.

## Figures and Tables

**Table 1:** Methods for preparation of polymer nanocomposites.

Method	Method to Obtain Nanocomposites	Results
In situ polymerization: emulsion and miniemulsion polymerization	dispersing fine nanofiller and nanoreinforcement in a monomer	monomers interact with the nanofiller surface and form a uniform suspension
Solution casting and latex method or solvent processing	1. filler dispersal in the polymer solution 2. solvent evaporation 3. freeze-drying and hot-pressing 4. freeze-drying, extruding and hot-pressing the mixture. 5. surfactant addition 6. grafting of long chains onto nanofiller surface	sandwiched multilayer structures forming of filler-rich layers polymer polarity-based results The dispersion of nanoparticles in the nanocomposite film strongly depends on the processing technique and conditions
Direct addition/extruder Blending, melt processing Shear mixing	1.addition of filler directly into the melted polymer 2. blending either by (mechanical mixer and extruder)	target: to obtain uniform distribution of the nanofillers in a polymer matrix
Deposition or layer (LBL) assembly	Layer-by-layer deposition	sequential substrate dipping in clay and polycation solutions were adopted to make the coating Multilayer films
Dispersion and chemical reaction	UV-curing in presence of photo-initiator; casting, evaporating the solvent	cross-linked nanocomposites
Electrospinning	development of electrospun nanofibers	nanofibres with different morphologies

**Table 2:** Some examples of nanofillers intended to be used in nanocomposites for food packaging (for many examples see also recent reviews on this subject [41,42,43,44].

Type of Nanofiller	Matrix	Preparation	Properties/Applications	Ref
Organoclay	LDPE and HDPE	melt mixing using PE grafted with maleic anhydride as compatibilizer-exfoliation	oxygen permeability of PE decreases gradually with the clay concentration, reaching a maximum reduction of ∼30% for 15 wt % MMT; dynamic moduli increase showing pseudo solid-like behaviour at clay concentrations higher than 8 wt %.	[45]
Nanolayers of Nanoter^TM^ from NanoBioMatters LTD Spain	PE	melt processing	very good barrier properties	[46]
4% MMT	EPDM	melt processing	decreased N_2_ permeability by 30%	[46]
Bentonite	PLA	solution casting	improve strength and modulus; decreased elongation at break	[47]
5% MMT	PVOH	casting	90% reduction in water permeability retaining optical clarity	[46]
MMT	proteins and polysaccharides	casting	60% reduction in water permeability	
1.1%–4%–10% Various unmodified and organically modified MMT, Cloisite 25A, Nanoter^TM^	PLA, PCL, PHA, PHBV Strach	monolayer packaging	reduction of oxygen and water permeability	
MMT	chitosan films	solvent casting	exfoliated and intercalated structure depending on MMT amount, Tensile strength of a chitosan film was enhanced and elongation-at-break decreased	[48]
MMT	poly (ε-caprolactone) (PCL)	electrospinning	improved mechanical properties even elongation at break	[49]
Anionic sodium MMT exfoliated	cationic polyacrylamide on a PET substrate	Layer-by-layer (LbL) self-assembly multilayer film	oxygen transmission rate (OTR) decreased as a function of number of bilayers deposited, until a negligible value–below 0.005 cm^3^/(m^2^ day atm)—for a 30-bilayer film, microwaveable and with a good optical transparency (higher than 90%), it was presented as a good candidate for aluminium foil replacement in food packaging.	[50]
5% Clay ZnO stabilized with sodium carboxymethylcellulose	thermoplastic starch; gelatinized starch film glycerol plasticized-PEA starch	melt extruded	improve the mechanical strength of biopolymers, decreased water vapour permeability by using only 5% (*w/w*) of clays; the highest exfoliation and best improvement in mechanical properties, exfoliated clay.	[51,52]
1 wt % and 5 wt % of modified (surfactant-modified) and un-modified cellulose nanocrystals,	PLA	solvent casting method in the presence of surfactant	reductions of 34% in water permeability for the cast films with 1 wt % of surfacant modified-CNC; good oxygen barrier properties; the migration level of the studied nano-biocomposites was below the overall migration limits required by the current normative for food packaging materials in both non-polar and polar simulants.	[53]
Up to 3% cellulose nanocrystals CNCs	PLA	extrusion, twin-screw extruder	water vapour permeability decreased gradually with increasing addition of CNCs up to 3%; good oxygen barrier properties; enhanced barrier and mechanical properties	[54]
Cellulosic nanoparticles in chloroform and layered silicates; whiskers	PLA	dispersion in non-aqueous medium, casting; addition of PVOH	An improvement in storage modulus over the entire temperature range for both nanoreinforcements together with shifts in the tanδ peaks for both nanoreinforcements to higher temperatures; reduction in the oxygen permeability for the bentonite nanocomposite but not for the MCC nanocomposite. The amount of light being transmitted through the nanocomposites was reduced compared to pure PLA indicating that both nanoreinforcements were not fully exfoliated	[47,55]
cellulose whiskers extracted from PEA hull fibres with different hydrolysis times, which resulted in different aspect ratios.	PEA starch matrix		The composite produced by using the whiskers with the highest aspect ratio exhibited the highest transparency and best tensile properties; enhances thermomechanical properties, reduces the water sensitivity and keeps biodegradability; Tg increases; moisture resistance improved	[56,57]
cellulose whiskers	PVOH	solution casting, water solvent	the modulus increased by orientation of reinforcement under magnetic field	[58]
cellulose nanowhiskers	k/l carrageenan	casting	high crystallinity, enhanced water barrier of carrageenan	[59]
	PLA	casting	improved barrier properties to gases and vapours, fully renewable biocomposites for biopackaging	
Bacterial cellulose nanowhiskers	EVOH or PLA	electrospinning	increased thermal stability	
Bacterial cellulose nanowhiskers	EVOH	melt compounding	enhanced barrier and mechanical properties of EVOH	
30 wt % of Straw cellulose whiskers	poly(styrene-*co*-butyl acrylate) latex film	freeze-drying and moulding a mixture of aqueous suspensions	modulus more than a thousand times higher than that of the bulk matrix	[60]
Aqueous suspensions of polysaccharide (cellulose, chitin or starch) nanocrystals tunicin (the cellulose extracted from a tunicate–a sea animal) whiskers, wheat straw or sugar beet cellulose nanocrystals, potato starch nanocrystal and squid pen and Riftia tubes chitin whiskers	hydrophobic polymers as: styrene and butyl acrylate [poly(S-*co*-BuA)] poly(β-hydroxyoctanoate) (PHO) polyvinylchloride (PVC), waterborne epoxy, natural rubber (NR) and polyvinyl acetate (PVAc), poly(styrene-*co*-hexyl-acrylate)	dispersion of these nanocrystals in non-aqueous media is possible using surfactants or chemical grafting long chain surface chemical modification; mixing and casting the two aqueous suspensions, freeze-drying and hot-pressing or freeze-drying, extruding and hot pressing; mixture extrusion methods, miniemulsion polymerization	films; larger latex particle size results in higher mechanical properties	[30]
aqueous suspension of polysaccharide nanocrystals	hydrosoluble or hydrodispersible polymers as: reinforced starch, silk fibroin, poly(oxyethylene), polyvinyl alcohol, hydroxypropyl cellulose, carboxymethyl cellulose or soy protein isolate	mixing and casting the aqueous solutions, freeze-drying and hot-pressing		
starch nanocrystals	waterborne polyurethane	solution casting; chemical grafting of starch nanocrystals	enhanced strength, elongation and Young’s modulus. The chemical grafting of the starch nanocrystals StNs did not affect positively the strength and elongation, because such a treatment inhibited the formation of physical interaction and increasing network density in nanocomposites	[61]
Coating of cotton and tunicin whiskers by a surfactant phosphoric ester of polyoxyethylene (9)-nonyl phenyl ether leads to stable suspensions in toluene and cyclohexane or chloroform	atactic polypropylene, isotactic polypropylene, or (EVA)	dispersion in non-aqueous medium, casting	decreased mechanical properties	[62,63]
Chitin whiskers	protein isolate thermoplastics	solution-casting technique	improved not only the tensile properties (tensile strength and elastic modulus) of the matrix but also its water resistance	[64]
chitin whiskers	chitosan films	solution-casting technique	improved chitosan films tensile strength until a whisker content of 2.96%, while higher increases of whiskers contents resulted in decreasing strength. Improved water resistance of the films.	[65]
chitosan–tripolyphosphate (CS–TPP) nanoparticles	hydroxypropyl methylcellulose (HPMC) films	solution-casting technique	improved mechanical and barrier properties of the films	[66]
carbon-based graphene, 20 to 60 nm in thickness and 0.5 to 25 μm in diameter, at 1 to 5 wt % loading	poly(methyl methacrylate) (PMMA)	dispersion at 30 °C by high speed shearing methods	heat resistant, high barrier nanocomposites promising in food packaging; increase the glass transition temperature of PMMA	[67]

**Table 3:** Formulations including antimicrobials as bioactive compounds intended to use in food packaging.

Active Agent	Substrate or Matrix	Technique	Characteristics/Observations	Ref.
**Antimicrobials**				
Essential oils; adhesion promotors (e.g., acrylic or vinyl resins or nitrocellulose) and fixatives	common packaging materials	different coating or spraying	controlled release	[193]
Bacteriocins (ex: Nisaplin^®^ nisin, pediocins); spice and herb extracts; Organic acids, microencapsulated nisin	oolycaprolactone, alginate, crosslinked chitosan/cellulose nanocrystal	crosslinking reaction under γ-irradiation	nanocellulose/PCL and alginate/cellulose nanocrystal based edible films and ready-to-eat meat	[194]
Silver nanoparticles AgNP	poly(3-hydroxybutyrate-*co*-3-hydroxyvalerate	extrusion/blending		[195]
Silver	poly(l-lactide)	melt-compounding	sustained release of antimicrobial silver ions in food applications	[196]
AgNP/peptide/cellulose nanocrystals	PLA	solvent casting	significant inhibition of microbial growth; migration rates below values reported by international imposed limits	[197]
AgNP/kaolinite	PLA and poly(butylene adipate-*co*-terephthalate)	blow films	the composite showed its biodegradation extent of 69.94% (after 90 days), offering good biodegradability for use as a material for the production of degradable plastic bags. The ageing, hydrolytic degradation and biodegradation of PLA-based films could be tailored by Ag kaolinite incorporation	[198]
AgNPs	multilayer films of PHBV3 and electrospun fibres	compression-moulding, 180 °C and 1.8 MPa for 5 min; coated with PHBVs and PHBVs/AgNsP ultrathin fibre mats produced by electrospinning followed by an annealing step; electrospun coating (~0.71 ± 0.01 mg/cm^2^)	multilayer materials for food packaging and food contact surface applications; efficient antimicrobial materials; bactericidal effect against *Salmonella enterica*	[191]
AgNP	PVOH	mixing a colloidal solution	improved thermal properties, enhancing stability and increasing Tg.	[199]
2 wt % Ag-NPs	polyamide 6	thermal reduction of silver ions during the melt processing of a PA6/silver acetate mixture	effective against E. coli	[200,201]
TiO_2_/Ag grafted with γ-aminopropyltriethoxysilane	PVC	mixing	good antibacterial properties by photocatalytic bacterial inactivation	[202]
nano-Ag 35%, nano-TiO_2_ 40%, kaolin 25%) (30%)	PE	high-speed mixer; extruded by a twin-screw extruder and then film was obtained	Ag-NPs retarded the senescence of jujube, a Chinese fruit.	[203]
ZnO:Cu/Ag	PLA	melt processing	Antimicrobial materials	[189]
cellulose nanocrystal/silver nanohybrids bifunctional nanofillers—10 wt % CNC-Ag	poly(3-hydroxybutyrate-*co*-3-hydroxyvalerate)	solution casting	high performance nanocomposites with improved thermal, mechanical and antibacterial properties against both *Gram-negative E. coli* and *Gram-positive S. Aureus* of PHBV. Reduced water uptake and water vapour permeability; lower migration level in both non-polar and polar simulants because of increased crystallinity and improved interfacial adhesion. Great potential applications in the fields of food, beverage packaging and disposable overwrap films.	[204]
30 μm thick coating containing 2–6% cinnamon essential oil	PP	spreading	controlled release, total inhibition *against Aspergillus* flavus and niger and *Penicillium roqueforti* and *Penicillium expansum*	[205]
4% thyme and oregano essential oils	corona treated LDPE	ionizing treatment and directly extrusion	controlled release, inhibit *Escherichia coli* 0157:H7, *Salmonella* Typhimurium and *Listeria monocytogenes*, changes in barrier properties	[206]
Organic-inorganic hybrid coatings, polyvinyl alcohol with improved water resistance	poly(ethylene terephthalate) and oriented polypropylene	sol-gel technique condensation of hydroxylated monomers and polymers into a network	multilayer materials for packaging applications	[207]
Antifungal agent natamycin was embedded in a tetraethyl orthosilicate/EVOH gel	plasma treated PLA films	sol-gel	antimicrobial coatings; controlled release; inhibit mould growth on cheese stored for 30 days	[208]
Nanosilver or chitosan	LDPE	melt-blended and layered deposited silver	silver ion migration from the nanocomposites into the food simulants and apple juice was less than the cytotoxicity-level concentration (10 mg kg^−1^) in all cases over 30 days	[209]
Antimicrobial photocatalysts (e.g., TiO_2_)	PLA multi-layered hybrid coatings	sol-gel technique	Controlled release, antimicrobial	[210]
Polydiacetylene liposomes containing cinnamaldehyde as liposome-encapsulated cinnamaldehyde	amine-functionalized silane monolayer on piranha treated glass or amine-functionalized PLA films	nanoencapsulation and immobilization of cinnamaldehyde	Controlled release, efficient against *Bacillus cereus*	[211]
Sorbic acid and/or lauric arginate ester and chitosan and nisin	PLA or corona treated PLA	directly coated with the solutions, or treated with solution-coated polylactic acid (PLA) films	2–3 logarithmic reductions of *Listeria innocua* (2–3 logarithmic reductions), *Listeria monocytogenes* and *Salmonella* Tiphymurium/negative effect on CO_2_ gas barrier properties. There was no significant difference in the effectiveness of antimicrobial films versus the coatings. Antimicrobial packaging may be used alone, or in combination with flash pasteurization, in preventing foodborne illness due to post processing contamination of ready-to-eat meat products.	[212,213]
Peptide nisin entrapped in polyethylene oxide brushes grown on silane modified silicon wafers which protected nisin.	polyethylene oxide	entrapment	inhibition of Gram positive bacterium *Pediococcus pentosaceous* over a period of seven days	[214]
Pullalan powder was rendered cationic by reaction with an amine terminated silane as 3-aminopropyltrimethoxysilane	antimicrobial pullulan	immobilization	inhibits *Staphylococcus aureus* and *Escherichia coli*	[215]
Enzyme lysozyme	covalently attached onto UV-ozone treated EVOH films	immobilization via carbodiimide chemistry	inhibits *Listeria monocytogenes*	[216]
(3-Bromopropyl)triphenylphosphonium	poly(butylene adipate-*co*-terephthalate) functionalized with a quaternary phosphonium compound, (3-bromopropyl) triphenylphosphonium	immobilization by azide-alkyne “click” reaction	inhibits *Escherichia coli*	[217]
SO_2_	multi-layered film made of PA and PE was subjected to atmospheric plasma treatment (Ar, Na_2_O and SO_2_) on the PA side of the films	immobilization	inhibits *Escherichia coli* (82%), *Staphylococcus aureus* (86%), *Listeria monocytogenes* (63%), *Bacillus subtilis* (79%) and *Candida albicans* (75%)	[218]
Chitosan (polycation) and κ-carrageenan (polyanion)	aminated PET	layer-by-layer assembly	improved gas barrier properties	[219,220]
Lysozyme: κ-carrageenan alternated with two layers of the antimicrobial enzyme lysozyme	amine-functionalized PET films	layer-by-layer assembly	improved oxygen and water vapour permeability	[221]

**Table 4:** Formulations including antioxidants and biocatalysts as bioactive compounds intended to use in food packaging.

**Antioxidants**				
Citrus oil	plasma treated PET trays	spray deposition of citrus oil in methanol	controlled release; antioxidant activity with cooked turkey meat and retained activity after six months of storage	[222,223]
Rosemary extract	LDPE plastic wrap or a polymeric carrier	applied direct onto LDPE plastic wrap or with a polymeric carrier	controlled release; 0.45 mg rosemary cm^−2^	[224]
α-Tocopherol	paperboard using a vinyl acetate-ethylene copolymer as a carrier for controlled release	solvent casting coating at a concentration of 3%	antimicrobial and antioxidant coating; controlled release	[225]
gallic acid	chitosan	immobilization by carbodiimide assisted reaction	reduced oxidation of peanuts	[226]
Metal oxide coatings Aluminium oxide or silicon oxide	biaxially oriented polypropylene and polyethylene terephthalate film substrates	reactive evaporation using an industrial high-speed vacuum deposition technique ‘boat-type’ roll-to-roll metallizer	transparent barrier coatings based on aluminium oxide or silicon oxide fulfil requirements such as product visibility, microwaveability or retortability reduce oxygen diffusion	[227,228]
Tannic acid and poly(allylamine hydrochloride)	glass slides	layer-by-layer assembly	the number of bilayers increases in overall scavenging activity	[229]
Caffeic acid	polypropylene packaging materials coating	photografting	prevent oxidative degradation of ascorbic acid in orange juice	[230]
Acrylic acid	metal chelating active packaging coatings PP films, PP-g-PAA	photografting	prevent lipid oxidation in food emulsions	[231,232,233]
Hydroxamic acid photografted polyhydroxamate chelators Plant-derived phenolic compounds, metal chelating	PP	*in situ* polymerization of a mixture of catechol and catechin and oxidative polymerization with laccase and in alkaline saline and photografting	surface adhesion properties upon polymerization; non-migratory iron chelating active packaging material biomimetic iron chelating active packaging material, inhibit oxidation of food	[234,235,236]
Lignosulfonate	Alginate	Solution casting	Antioxidant and UV protective films	[113]
**Biocatalysts**				
Lactase blended into polyethylene oxide nanofibers	oxygen scavenging	electrospinning-enzymes or other active agents are incorporated into polymer nanofibers	controlled release, retained up to 93% of free enzyme activity	[237,238]
Lactase	attachment of lactase to polyethylene films;	immobilization β-galactosidase bound to amine-functionalized PE films by a dialdehyde tether; polyethylene glycol tether size influences the attachment	reduce milk lactose in package; retained activity of immobilized lactase	[239,240]
Lactase conjugated to nanomaterials	lactase immobilization onto nanostructures	lactase was attached to carboxylic acid functionalized magnetic nanoparticles 18 nm, 50 nm and 200 nm in diameter using carbodiimide chemistry.	retained activity of immobilized lactase; reducing the particle size of magnetic nanoparticles can increase the activity retention of conjugated lactase	[241]
Lactase and polyethylenamine, glutaraldehyde	lactase covalently bound to low-density polyethylene	layer-by-layer assembly	more enzyme is immobilised but diffusion is difficult	[242]
Glucose oxidase	electrospun polyvinyl acetate/chitosan/tea extract fibres	electrospinning	reduce oxygen in packaged foods	[243]
Glucose oxidase	chitosan	LbL films	biosensors	[244]
Oxalate oxidase, oxygen-reducing enzymes in coatings and films for active packaging	extrusion-coated liner of polypropylene on top of the coating.	entrapment in a latex polymer matrix	protective packaging gas carbon dioxide; oxygen scavenging in active packaging; retained catalytic activity through entrapment in a latex polymer matrix	[245]
Laccase-catalysed reduction of oxygen	it was possible to use lignin derivatives as substrates for the enzymatic reaction.	laccase-catalysed reaction created a polymeric network by cross-linking of lignin-based entities,	resulted in increased stiffness and water-resistance of biopolymer films	[238]
Catalase	layered haemoglobin, PS	Immobilization	create a physical and chemical protective barrier	[229,246]
Fungal naringinase	cross-linking naringinase to polyvinyl alcohol and alginate	immobilization	bitterness reduction in grapefruit juice	[247,248]
Protease, trypsin and endoproteinases	PP, PVOH and PS	photografting, carboiimide chemistry -covalently couple enzymes (via amine groups) to carboxylic acid groups poly(ethylene) glycol methacrylate and 4-vinyl-2,2-dimethylazolactone	antibody analysis in enzyme reactors	[249]
Hydrolase urease	photografted polytetrafluoroethylene	photografting	remove urea from beverages and foods	[250]
Glucose oxidase catalase for oxygen scavenging activity	low-density polyethylene and paper board multilaminate or combinations of LDPE, PP and PLA	industial lamination	scaled-up production in tetra pack pilot plan, scavenge oxygen to improve food shell life	[251]
Polysiloxane-based healing agents	acrylate matrix	polymer coatings coaxial electrospinning	self-healing polymer coating systems	[252]
